# The inflammatory cytokine IL-6 induces FRA1 deacetylation promoting colorectal cancer stem-like properties

**DOI:** 10.1038/s41388-019-0763-0

**Published:** 2019-02-25

**Authors:** Tingyang Wang, Ping Song, Tingting Zhong, Xianjun Wang, Xueping Xiang, Qian Liu, Haiyi Chen, Tian Xia, Hong Liu, Yumiao Niu, Yanshi Hu, Lei Xu, Yingkuan Shao, Lijun Zhu, Hongyan Qi, Jing Shen, Tingjun Hou, Riccardo Fodde, Jimin Shao

**Affiliations:** 1 Department of Pathology & Pathophysiology, and Cancer Institute of the Second Affiliated Hospital, Zhejiang University School of Medicine, Hangzhou, China; 2 Key Laboratory of Disease Proteomics of Zhejiang Province, Key Laboratory of Cancer Prevention and Intervention of China National Ministry of Education, and Research Center for Air Pollution and Health, Zhejiang University School of Medicine, Hangzhou, China; 3 College of Pharmaceutical Sciences, Zhejiang University, Hangzhou, China; 4 Department of Bioinformatics, College of Life Sciences, Zhejiang University, Hangzhou, China; 5 Institute of Bioinformatics and Medical Engineering, School of Electrical and Information Engineering, Jiangsu University of Technology, Changzhou, China; 6 Key Laboratory of Precision Diagnosis and Treatment for Hepatobiliary and Pancreatic Tumor of Zhejiang Province, First Affiliated Hospital, Zhejiang University School of Medicine, Hangzhou, China; 7 Department of Pathology, Erasmus MC Cancer Institute, Erasmus University Medical Center, Rotterdam, The Netherlands; 8 Present Address: Department of Pathology, Sir Run Run Shaw Hospital, Zhejiang University School of Medicine, Hangzhou, China; 9 Present Address: Zhejiang Normal University-Jinhua People’s Hospital Joint Center for Biomedical Research, Jinhua, China

**Keywords:** Cancer stem cells, Colorectal cancer, Acetylation, Cytokines

## Abstract

Colorectal cancer (CRC) has long been known for its tight association with chronic inflammation, thought to play a key role in tumor onset and malignant progression through the modulation of cancer stemness. However, the underlying molecular and cellular mechanisms are still largely elusive. Here we show that the IL-6/STAT3 inflammatory signaling axis induces the deacetylation of FRA1 at the Lys-116 residue located within its DNA-binding domain. The HDAC6 deacetylase underlies this key modification leading to the increase of FRA1 transcriptional activity, the subsequent transactivation of *NANOG* expression, and the acquisition of stem-like cellular features. As validated in a large (*n* = 123) CRC cohort, IL-6 secretion was invariably accompanied by increased FRA1 deacetylation at K116 and an overall increase in its protein levels, coincident with malignant progression and poor prognosis. Of note, combined treatment with the conventional cytotoxic drug 5-FU together with Tubastatin A, a HDAC6-specific inhibitor, resulted in a significant in vivo synergistic inhibitory effect on tumor growth through suppression of CRC stemness. Our results reveal a novel transcriptional and posttranslational regulatory cross-talk between inflammation and stemness signaling pathways that underlie self-renewal and maintenance of CRC stem cells and promote their malignant behavior. Combinatorial treatment aimed at the core regulatory mechanisms downstream of IL-6 may offer a novel promising approach for CRC treatment.

## Introduction

Tumor-promoting inflammation is emerging as one of the main hallmarks of cancer [1] and colorectal cancer (CRC) best exemplifies the tight link between inflammation, tumor onset, and malignant progression [2]. We and others have previously shown that interleukin-6 (IL-6) represents a key inflammatory cytokine and that its downstream effector STAT3 (signal transducer and activator of transcription 3) underlies CRC proliferation, epithelial-to-mesenchymal transition (EMT) [3], tumorigenesis [4], and stemness [5]. In hepatocellular carcinoma (HCC), tumor-associated macrophages promote CD44^+^ stem-like cell expansion, and IL-6/STAT3 signaling inhibition using Tocilizumab (TCZ) inhibits this expansion [5]. However, the downstream effectors of the IL-6/STAT3 signaling axis that underlie cancer stem cell (CSC) properties are yet unknown.

Conventional cytotoxic cancer treatment is only partly effective because it mainly targets the bulk of cancer cells without affecting the stem-like cancer cells capable of tumor initiation, self-renewal, differentiation, and chemoresistance. The latter highlights the need to circumvent therapy resistance by specifically targeting the stem-like cellular component of malignancies. Thus far, CD44, CD133, and CD166 were shown to represent informative CSC markers in colon cancer [6–9]. Moreover, it has been demonstrated that NANOG, a core homeobox transcription factor (TF) playing key roles in the maintenance and preservation of self-renewal in embryonic stem cells (ESCs), is also expressed in CSCs from different tumor types, including liver and colon cancers [10–13].

FRA1 is a member of the FOS family of TFs encoded by the *FOSL1* gene and an important transcriptional downstream target of the IL-6/STAT3 signaling axis leading to CRC aggressiveness through EMT induction [3]. Accordingly, FRA1 is highly expressed in multiple cancers and is thought to play key roles in neoplastic transformation [14], motility [15], cancer drug addiction [16], and stemness [17, 18]. Elevated *FOSL1* (FRA1) expression level was reported to result from the activation of the IL-6/STAT3 [3], RAS-RAF-MEK-ERK-RSK [14, 15], and PKCα/θ [17, 19] pathways both at the transcriptional and posttranslational levels. In the latter case, phosphorylation of four C-terminal residues, namely, Ser-252, Ser-265, Thr-223, and Thr-230, inhibits FRA1 degradation [15, 19].

Acetylation is a well-known regulatory posttranslational modification. In particular, acetylation at specific residues of several TFs has been shown to represent an important regulatory mechanism. Notably, lysine acetylation is not only restricted to histones but is also found in numerous TFs, including p53, nuclear factor (NF)-κB, and STAT3 [20]. Mechanistically, TF acetylation leads to changes in protein–protein and protein–DNA interaction [21–23], thus resulting in a plethora of downstream effects including increased/decreased transcription, protein stabilization, steric prevention of ubiquitination, and chromatin remodeling.

In the present study, we investigated posttranslational regulatory mechanisms downstream of the IL-6/STAT3/FRA1 inflammatory signaling axis that mediate colon cancer stemness and malignancy and explored novel combinatorial therapeutic approaches to target CRC stem and bulk cells.

## Results

### IL-6 promotes colon cancer stemness in an FRA1-dependent manner

In colon cancer, IL-6 is known to be secreted by stromal fibroblasts, several types of immune cells, and by parenchymal cancer cells to activate STAT3 signaling, thereby mediating tumor-promoting effects [4]. In view of this, we first excluded autocrine IL-6 secretion in the two CRC cell lines employed in this study, namely, DLD1 and HT-29, by enzyme-linked immunosorbent assay. As shown in Figure S1a, almost no IL-6 was detected in either cell line, independently of *FOSL1* expression. Next, we determined the effects exerted by recombinant IL-6 on both cell lines. Phenotypic analyses by in vitro cell migration, invasion, sphere formation, and chemo-resistance assays and by in vivo lung metastasis assay in nude mice consistently indicated that IL-6 exposure promoted CRC stemness and malignancy (Figure S1b-S1e).

**Fig. 1:**
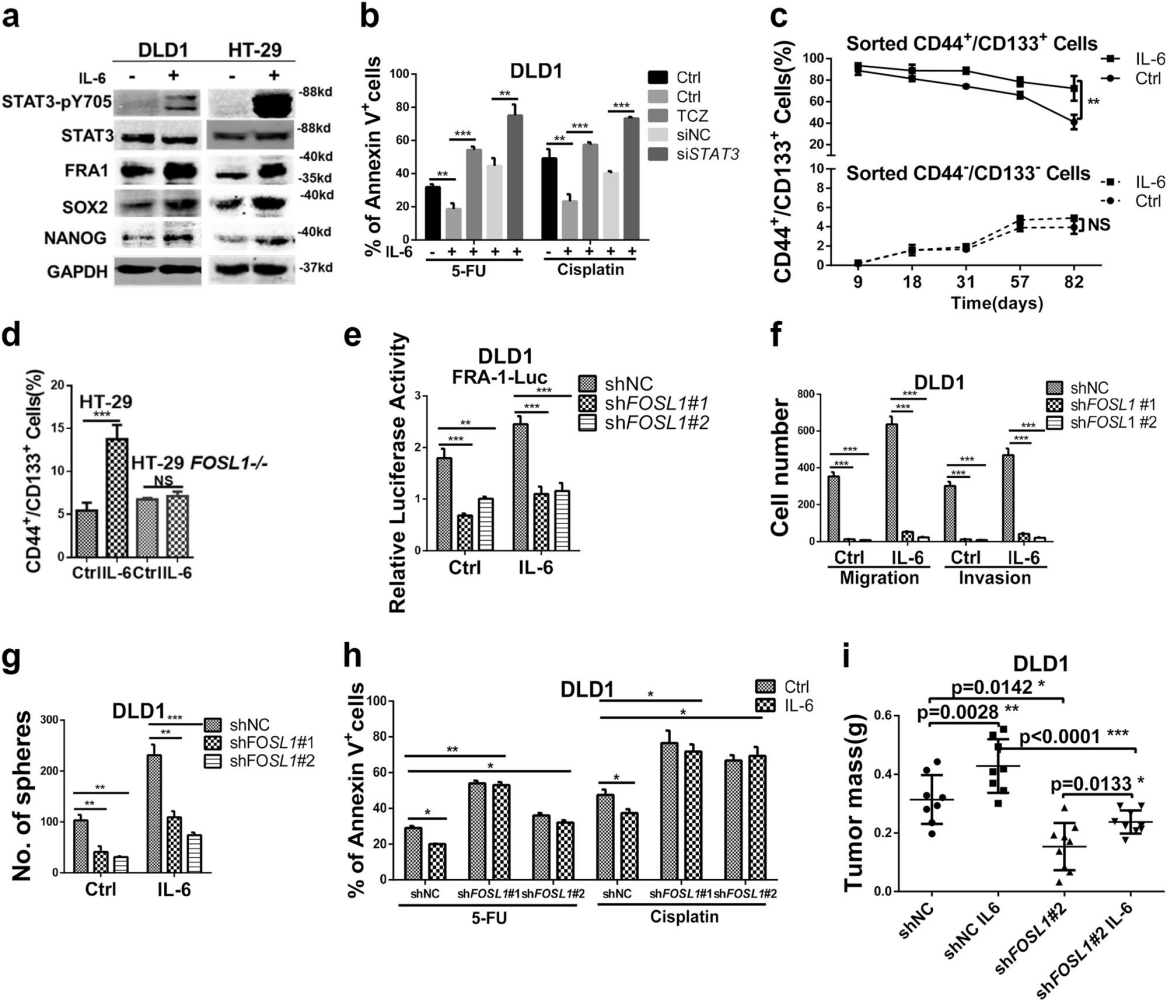
Interleukin (IL)-6 promotes colon cancer stemness in an FRA1-dependent manner. **a** Western blot analysis of DLD1 and HT-29 cells cultured in the presence/absence of 50 ng/ml IL-6 for 24 h. Protein levels of STAT3-pY705, STAT3, FRA1, SOX2, NANOG, and GAPDH were examined. **b** DLD1 cells were cultured in medium supplemented with the chemotherapeutic drugs 5-Fluorouracil (5-FU) and cisplatin and in the presence/absence of IL-6 (50 ng/ml), Tocilizumab (5 μg/ml), and si*STAT3*. The percentage of apoptotic (Annexin V^+^) cells were employed as a measure of sensitivity to the treatment when compared to the controls. **c** CD44^+^/CD133^+^ and CD44^−^/CD133^−^ DLD1 cells were sorted by fluorescence-activated cell sorter (FACS) and cultured in the presence/absence of IL-6 for the indicated times. These cultures were monitored at the indicated time points by FACS analysis. Quantification of CD44^+^/CD133^+^ cells in triplicate (mean ± SD) are depicted. **d** CD44/CD133 FACS analysis of parental (*FOSL1*^+/+^) and *FOSL1*^−/−^ HT-29 cells cultured in the presence/absence of IL-6 for 7 days. The relative percentage of the CD44^+^/CD133^+^ subpopulation is indicated in the histogram. **e** FRA1 transcriptional activity was measured in shNC, sh*FOSL1*#1, and sh*FOSL1*#2 DLD1 cells cultured in the presence/absence of IL-6 (50 ng/ml). **f**, **g** Migration and invasion assays (**d**) and sphere formation assays (**e**) were performed with non-target control (shNC), sh*FOSL1*#1, and sh*FOSL1*#2 DLD1 cells cultured in the presence/absence of IL-6. Results are shown as histograms showing quantitative values of the number of cells (or spheres) from triplicate experiments (mean ± SD). **h** shNC and *FOSL1* knockdown (sh*FOSL1*#1 and sh*FOSL1*#2) DLD1 cells were cultured in medium supplemented with the chemotherapeutic drugs 5-FU and Cisplatin and in the presence/absence of IL-6 (50 ng/ml). The percentage of apoptotic (Annexin V^+^) cells indicated that both sh*FOSL1*#1 and sh*FOSL1*#2 were characterized by increased sensitivity when compared to the shNC control cells. **i** 5 × 10^5^ sh*FOSL1*#2 and, as a control, shNC DLD1 cells were cultured in the presence/absence of IL-6 for 5 days and then injected subcutaneously into the flanks of BALB/C nude mice. DLD1 cells treated with IL-6 increased tumor mass when compared with the shNC group. And this effect was attenuated upon *FOSL1* knockdown. *p < 0.05, **p < 0.01, ***p < 0.001. Unpaired *t* test. Data are presented as mean ± SD

Previously, we reported that IL-6-activated STAT3 upregulates transcription of the *FOSL1* gene by directly binding to its promoter and further promoting CRC malignant progression through EMT activation [3]. As shown by western blot analysis in Fig. 1a, IL-6 stimulation resulted in STAT3 pathway activation and up-regulation of FRA1, SOX2, and NANOG expression in both DLD1 and HT-29 cell lines (Fig. 1a). Accordingly, inhibition of the IL-6/STAT3/FRA1 inflammatory signaling axis by anti-human IL-6R TCZ and by small interfering RNA (siRNA) knockdown of *STAT3* significantly reduced sphere-formation capacity and chemo-resistance of DLD1 cells upon IL-6 stimulation (Figure S1f and S1g; Fig. 1b).

To determine the effects of IL-6 on cancer stem cells, we measured by flow cytometry the expression of two colorectal CSCs markers, namely, CD44 and CD133 [6, 7]. Upon IL-6 treatment, the relative proportion of the CD44^+^/CD133^+^ CSC subpopulation increased from 3.3% to 13.8% and from 4.4% to 15.7% in DLD1 and HT-29, respectively (Figure S1h). Next, we sorted by fluorescence-activated cell sorter (FACS) the CD44^+^/CD133^+^ and CD44^−^/CD133^−^ subpopulations from IL-6-treated DLD1 cells. Sphere-formation and subcutaneous transplantation assays indicated that CD44^+^/CD133^+^ cells were characterized by increased self-renewal in vitro and tumor-propagating capacity in vivo when compared with CD44^−^/CD133^−^ cells (Figure S1i and S1j). Of note, IL-6 treatment enhanced these features in both subpopulations.

Next, we cultured the sorted cells in the presence/absence of IL-6 and followed their behavior by FACS analysis for 82 days. As depicted in Fig. 1c and Figure S1k, CD44^+^/CD133^+^ DLD1 cells were maintained at a significantly higher percentage under constant IL-6 stimulation, thus confirming its positive effect on the maintenance of the CSC subpopulation.

To assess whether IL-6 affects CSC properties through FRA1, we stably overexpressed the *FOSL1* gene in the DLD1 and HT-29 cell lines (Figure S2a). Increased *FOSL1* expression enhanced cell features including cell migration and invasion (Figure S2b) and sphere formation (Figure S2c). Accordingly, subcutaneous transplantation of *FOSL1-*overexpressing DLD1 cells into nude mice revealed increased tumorigenicity when compared with the empty vector (EV) group, and IL-6 stimulation enhanced this effect in EV group (Figure S2d).

To further study the role played by *FOSL1*/FRA1 in IL-6-driven colon cancer stemness, *FOSL1* gene knockout HT-29 cells were constructed by TALEN technology. In *FOSL1*^−/−^ HT-29 cells, the IL-6-induced expansion of the CD44^+^/CD133^+^ subpopulation was completely abolished, thus reinforcing the relevance of the IL-6/FRA1 signaling axis in regulating CSCs (Fig. 1d). Moreover, we knocked down *FOSL1* (sh*FOSL1*#1 and sh*FOSL1*#2) in DLD1 and HT-29 cells using a lentivirus-based approach (Figure S2e). *FOSL1* knockdown caused a significant impairment of FRA1 transcriptional activity as measured by the FRA1-Luc reporter activity in the presence/absence of IL-6 in the culture medium (Fig. 1e). Moreover, the positive in vitro effects of IL-6 on migration, invasion, and sphere formation were dramatically reduced by FRA1 downregulation (Fig. 1f, g; Figure S2f and S2g). In addition, *FOSL1* knockdown suppressed the IL-6-induced increase in chemo-resistance to 5-Fluorouracil (5-FU) and Cisplatin in DLD1 and HT-29 cells (Fig. 1h; Figure S2h). Consistent with the above in vitro findings, *FOSL1* knockdown also attenuated the tumor-enhancing effect of IL-6 in nude mice (Fig. 1i).

Overall, these results indicated that activation of the IL-6/STAT3/FRA1 inflammatory signaling axis promotes CRC stemness and progression toward malignancy.

### IL-6/STAT3-driven FRA1 Lys-116 deacetylation increases its transcriptional activity

**Fig. 2:**
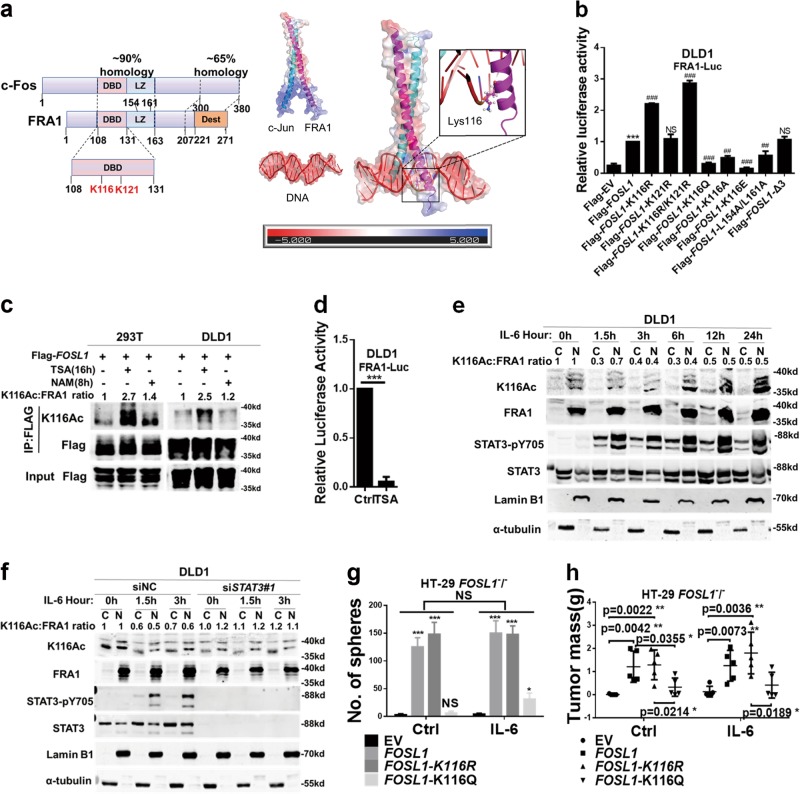
Interleukin (IL)-6/signal transducer and activator of transcription 3 (STAT3)-driven FRA1 Lys-116 deacetylation increases its transcriptional activity. **a** Left panel: schematic diagram of the homology between structural domains in c-Fos and FRA1. The highest homology is found in the DNA-binding domain that encompasses the two FRA1 acetylated lysine residues (K116 and K121) identified by mass spectrometry. Two leucine residues (L154 and L161) located in the Leucine zipper domain (LZ) were responsible for FRA1 heterodimerizing with the JUN family. Phosphorylation of many serine (S) or threonine (T) residues of FRA1 in destabilizer domain (DEST) increase its stability. Right panel: Charge density map of AP-1(c-Jun and FRA1), DNA and the complex. Lys116 in FRA1 chain is shown as sticks and spheres. **b** Relative luciferase activity in DLD1 cells co-transfected with wild-type and (deletion and point) mutant *FOSL1* constructs, together with pRL-TK as an internal control reporter. Δ3 deletion and L154A/L161A mutant constructs were here employed as controls (*p < 0.05, **p < 0.01, ***p < 0.001, compared with the Flag-EV; ^##^*p* < 0.01, ^###^*p* < 0.001, compared with the Flag-*FOSL1*). **c** Immunoprecipitation (IP) analysis of FRA1 K116 acetylation. The Flag-*FOSL1* construct was transfected into 293T and DLD1 cells, followed by treatment with the deacetylase inhibitors trichostatin A(TSA) or nicotinamide, and IP with antibodies directed against Flag. FRA1 acetylation was analyzed by western blot with the K116Ac-specific antibody. **d** FRA1 transcriptional activity in response to TSA treatment for 16 h and then measured by FRA1-Luc luciferase assay in DLD1 cells. **e** DLD1 cells were cultured in the presence of IL-6 for the indicated times and the cytoplasmic and nuclear protein fractions were separated for western blot analysis with the indicated antibodies. K116 acetylation was detected with the K116Ac antibody and normalized against the FRA1 protein. (LaminB1 and α-tubulin were employed as nuclear and cytoplasmic protein control, respectively). **f** DLD1 cells were transfected with siNC or *STAT3* small interfering RNAs and incubated with IL-6 for the indicated times. Cytoplasmic and nuclear protein fractions were separated for western blot analysis using the indicated antibodies. K116 acetylation was detected with K116Ac antibody and normalized against the FRA1 protein. **g**, **h** HT-29 *FOSL1*^−/−^ cells were constructed by TALEN and transfected with expression constructs encompassing wild-type and mutated *FOSL1*. These cells were then analyzed for their capacity to form spheres in vitro (**g**) and to form subcutaneous tumors in vivo upon IL-6 stimulation (**h**). *p < 0.05, **p < 0.01, ***p < 0.001. Unpaired *t* test. Data are presented as mean ± SD

Acetylation of non-histone proteins represents a key transcriptional regulatory event [20–23]. In order to elucidate the mechanisms of FRA1 acetylation and its functional consequences, a pan acetyl-lysine antibody (Ace-Lys) was employed. Immunoprecipitation (IP) analysis of ectopically expressed full-length and truncated (Flag- and Myc-tagged) FRA1 in 293T cells showed that the CREB-binding protein (CBP) can acetylate FRA1 and that the acetylated sites were located in the NH_2_-terminal 130 residues of FRA1 (Figure S3a and S3b). According to an online prediction tool [24], the K116 and K121 residues represent potential FRA1 acetylation sites within the NH_2_-terminal fragment. Mass spectrometric analysis of DLD1 cells confirmed that both Lys residues were indeed acetylated by CBP (Figure S3c). Of note, these residues were localized within the FRA1 DNA-binding domain (DBD), highly homologous to the corresponding domain in c-FOS. We then generated a structure model of the analogous c-Jun/FRA1 complex bound to the promoter motif showing that the K116 residue was in direct contact with DNA (Fig. 2a). However, alignment of the FRA1 and c-FOS sequences around the K116Ac target region revealed the presence of a key difference, namely the L117 residue in FRA1 corresponding to M149 in c-FOS (Figure S3d). In agreement with these in silico predictions, IP analysis of ectopically expressed Flag-EV, Flag-c-Fos and Flag-FRA1 in DLD1 cells showed that K116Ac antibody could hardly recognize c-Fos compared with FRA1 (Figure S3e). As shown in Figure S3f, the DBD of FRA1 and FRA1-K116R reached stability during the last 50 ns of molecular dynamics (MD) simulations, while the DBD of FRA1-K116Q fluctuated during the entire MD simulation, indicating that the DBDs of FRA1 and FRA1-K116R are more stable than that of FRA1-K116Q upon interaction with the target DNA domain. The protein–DNA binding free energies for the three complexes were estimated by MM/GBSA, and the binding affinities for COM, COM-K116Q, and COM-K116R were −189.97, −187.81, and −194.23 kcal mol^−1^, respectively (Table S1), indicating that the mutation of K116 to Gln weakened the binding of FRA1 to DNA, while the mutation of K116 to Arg strengthened the binding. To clarify the effect of the mutations on the protein–DNA binding quantitatively, the MM/GBSA free energy decomposition was performed. The energy contributions of the residues at position 116 for the DNA binding in the three systems were quite different (K116 = –5.41 kcal mol^−1^, Q116 = –4.7 kcal mol^−1^, and R116 = –6.87 kcal mol^−1^). As for the residue116, the difference was mainly contributed from the polar energy terms (∆*E*_ele_ + ∆*G*_GB_ = –1.67, –0.61, and –2.73 kcal mol^−1^ for K116, Q116, and R116, respectively) (Figure S3g).

Mutagenesis and luciferase reporter assays provided experimental confirmation of the above results: the FRA1-K116R mutant that mimics deacetylation at the Lys-116 residue was characterized by an increment of FRA1 reporter luciferase activity, whereas the acetylation mimic FRA1-K116Q/E/A mutants resulted in a reduction of FRA1-luc activity. Notably, the FRA1-K116R/K121R double mutant had similar effects when compared with the single FRA1-K116R mutant, while the K121R mutation alone did not affect FRA1 reporter activity. Besides, both the Δ3 deletion mutant (deletion of 3 C-terminal residues) and the dimerization-defective (L154A/L161A) mutant of FRA1 [25] did not affect FRA1-luc activity (Fig. 2b). Furthermore, additional experimental evidence excluded that the K116 and K121 residues of FRA1 were involved in protein stability and in its interaction with c-Jun (Figure S4a and S4b).

To enable the analysis of K116 deacetylation in endogenous FRA1, we generated a polyclonal antibody specifically recognizing the acetylated Lys-116 residue. The specificity of the anti-K116Ac antibody was validated by western blot and IP analyses (Figure S4c and S4d). Additional western blot analysis with the K116Ac antibody showed an increase of approximately 2–3-fold in K116 acetylation by trichostatin A (TSA; an inhibitor of histone deacetylase HDAC family I, II, and IV) but not by nicotinamide (NAM; an inhibitor of the SIRT family deacetylase), in both 293T and DLD1 cells (Fig. 2c). TSA treatment did reduce FRA1 luciferase activity (Fig. 2d). Hence, these results indicate that K116 deacetylation plays a key role in the regulation of FRA1 transcriptional activity.

Since IL-6/STAT3 signaling positively affects *FOSL1* transcription and thereby increases CRC stemness as shown above, we asked whether FRA1 K116 deacetylation could be directly involved in the modulation of CSCs properties. As shown in Fig. 2e, FRA1 mainly localizes to the nucleus of CRC cells in agreement with its function as a TF. After a short (0–3 h) exposure to IL-6, K116 acetylation level readily decreased while the overall expression level of FRA1 remained unchanged. Prolonged IL-6 treatment (6–24 h) further increased *FOSL1* transcription, though with a decrease in the relative level of K116-acetylated FRA1 versus total FRA1 in both nucleus and cytoplasm (Fig. 2e). Accordingly, siRNA-mediated *STAT3* knockdown markedly attenuated the IL-6-induced *FOSL1* expression and K116 deacetylation (Fig. 2f). Moreover, in vitro and in vivo assays showed that cells expressing wild-type FRA1 or the deacetylation mimic K116R were characterized by a significant increase in sphere formation and tumorigenic potential when compared with the K116Q acetylation mimic construct (Fig. 2g, h).

Taken together, these data showed that activation of the IL-6/STAT3 signaling axis increases FRA1 level not only by its transcriptional upregulation but also by posttranslational deacetylation at its K116 site, thus promoting colorectal CSC properties.

### HDAC6 deacetylates FRA1 and underlies its transcriptional activation downstream of IL-6/STAT3

**Fig. 3:**
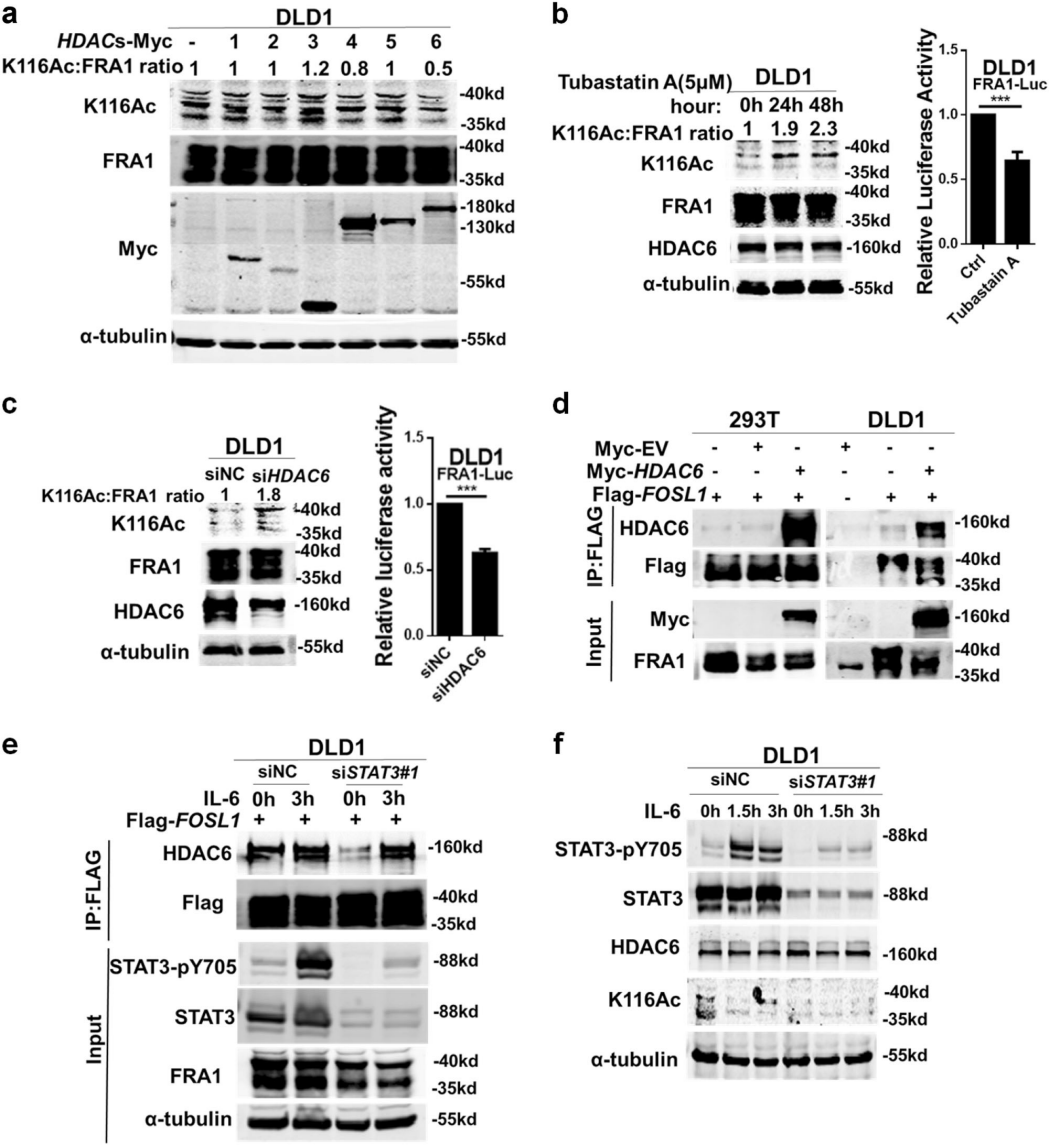
HDAC6 deacetylates FRA1 and underlies its transcriptional activation downstream of interleukin (IL)-6/signal transducer and activator of transcription 3 (STAT3). **a** Overexpression of *HDAC6*, but not of other *HDAC*s, decreases FRA1 acetylation levels. Each of the Myc-tagged *HDAC* (1–6) were transfected into DLD1 cells and the K116 FRA1 acetylation levels were determined by western blotting with the K116Ac antibody and normalized against the FRA1 protein. **b** Western blot analysis and relative FRA1-luc reporter activities in DLD1 cells treated with the HDAC6 small inhibitor Tubastatin A for the indicated times. **c** Small interfering RNA (siRNA) oligonucleotides directed against *HDAC6* were employed to transfect DLD1 cells. The levels of endogenous K116 acetylation, FRA1, and HDAC6 expression were analyzed by western blot. And the FRA1-luc reporter activity was assayed. **d** IP-Flag analysis of the interaction between FRA1 and HDAC6 in 293T and DLD1 cells transfected with the indicated plasmids. **e** DLD1 cells were transfected with Flag-*FOSL1* and the indicated siRNAs, followed by IL-6 stimulation for 3 h. IP-Flag analysis of HDAC6 was performed to assess the role of the IL-6/STAT3 signaling pathway on the FRA1–HDAC6 interaction. **f** DLD1 cells were transfected with non-target control oligonucleotides (siNC) or *STAT3* siRNAs and incubated with IL-6 for the indicated times. The levels of STAT3-pY705, STAT3, HDAC6, α-tubulin, and endogenous K116 acetylation protein were determined by western blot. ***p < 0.001. Unpaired *t* test. Data are presented as mean ± SD

Treatment with the HDAC inhibitor TSA, though not with the SIRT inhibitor NAM, increased K116 acetylation (Fig. 2c), thus indicating that HDACs may underlie FRA1 deacetylation. To provide experimental evidence for this, we first analyzed endogenous *HDAC*1–6 levels in the colon cancer cell lines DLD1, HT29, and 293T and showed that all six deacetylases are expressed albeit at different levels (Figure S5a). Next, we expressed *HDAC*1–6 individually in DLD1 cells. As shown in Fig. 3a, only HDAC6, an HDAC family II deacetylase that mainly targets non-histone proteins, decreased the K116 acetylation level of endogenous FRA1 in CRC cells. Moreover, treatment with Romidepsin (FK228; HDAC1/2 inhibitor) or Entinostat (MS-275; HDAC1/3 inhibitor) had no evident impact on K116 acetylation (Figure S5b). Accordingly, treatment with the specific HDAC6 inhibitor Tubastatin A increased K116 acetylation and decreased *FOSL1* luciferase activity (Fig. 3b). Similar results were obtained by the *HDAC6* knockdown in DLD1 cells (Fig. 3c). In agreement with these results, western blot analysis revealed that HDAC6 is localized in both the nucleus and cytoplasm of CRC cells (Figure S5c).

We then examined the physical interaction between HDAC6 and FRA1 by co-IP analysis. Both ectopic and endogenous HDAC6 could be coprecipitated by FRA1 in 293T and DLD1 cells (Fig. 3d, e). Moreover, this interaction was enhanced by IL-6 and attenuated by si*STAT3* (Fig. 3e), thus indicating that IL-6/STAT3 signaling may positively affect the interaction of HDAC6 with FRA1 and facilitate its deacetylation. Last, siRNA-mediated *STAT3* knockdown markedly attenuated IL-6-induced K116 deacetylation but had no effect on global *HDAC6* expression (Fig. 3f).

Overall, these results indicated that HDAC6 underlies K116 deacetylation of FRA1 and its transcriptional activation downstream of IL-6/STAT3.

### FRA1 trans-activates *NANOG* expression upon K116 deacetylation

**Fig. 4:**
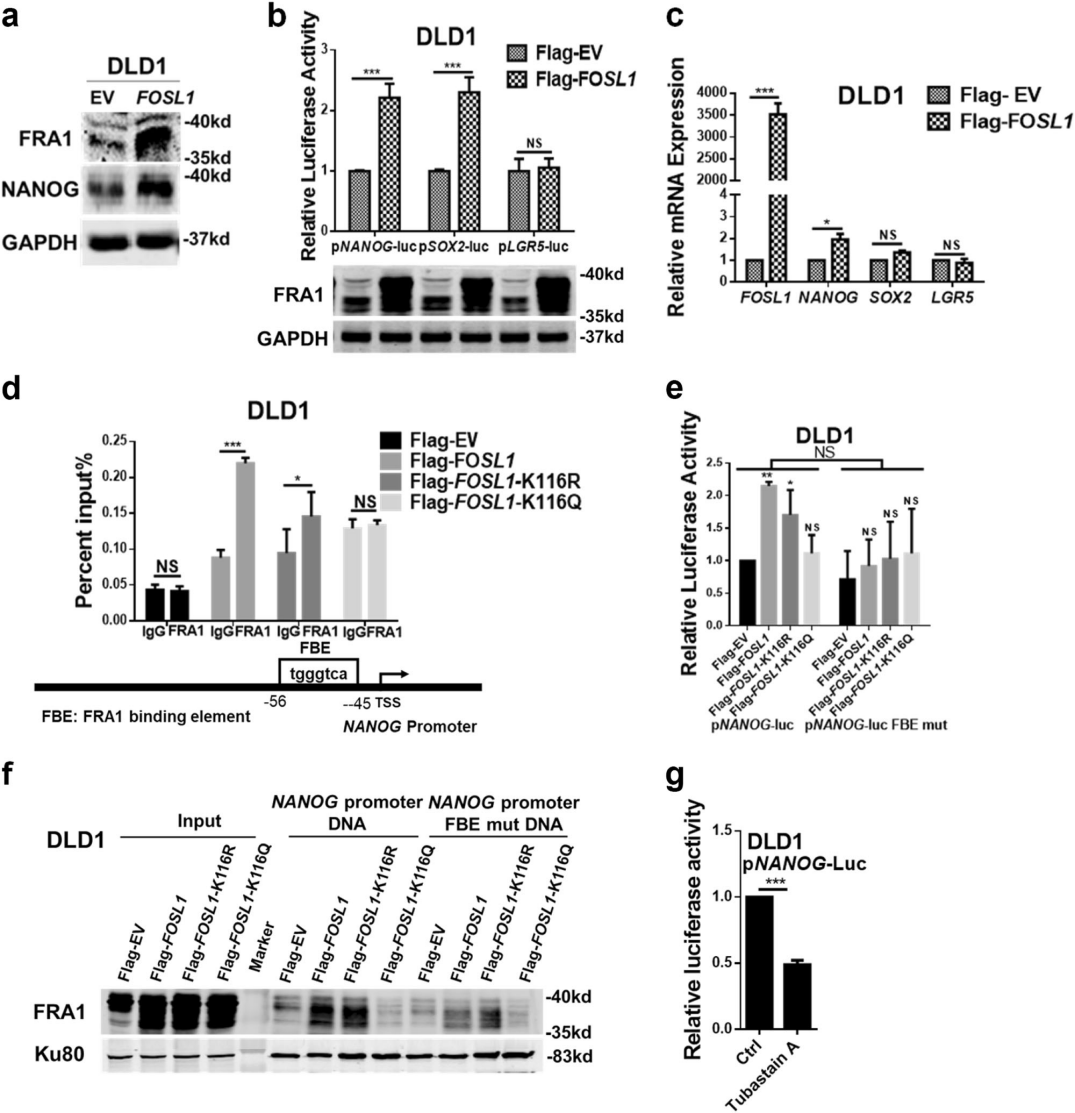
FRA1 trans-activates *NANOG* expression upon K116 deacetylation. **a** FRA1 and NANOG expression analysis by western blot in empty vector (EV) and *FOSL1*-overexpressing DLD1 cells. **b**, **c** Relative reporter luciferase activity (**b**) and mRNA levels (**c**) of *NANOG*, *SOX2*, and *LGR5* in EV and *FOSL1*-overexpressing DLD1 cells. **d** Chromatin immunoprecipitation (ChIP) analysis of DLD1 cells transfected with Flag-tagged wild-type and mutated *FOSL1* expression vectors. ChIP was performed with the Flag antibody. Quantitative reverse transcriptase–polymerase chain reaction analysis was performed on immune-precipitated DNAs using a primer pair specific for the *NANOG* promoter. **e** Relative luciferase activity of wild-type and FBE-mutant (predicted FRA1-binding site) *NANOG*-luc reporters in DLD1 cells transfected with wild-type and mutant *FOSL1* expression constructs. **f** DNA pull-down assay analysis of FRA1-binding ability to the biotin-labeled wild-type and FBE-mutant *NANOG* promoter probe (−404/−1) in DLD1 cells stably overexpressing wild-type and mutant *FOSL1*. Ku80 served as a control. **g** Relative luciferase activity of the *NANOG*-Luc reporter in DLD1 cells upon Tubastatin A treatment for 8 h. *p < 0.05, **p < 0.01, ***p < 0.001. Unpaired *t* test. Data are presented as mean ± SD

To further explore direct transcriptional targets of FRA1 in the regulation of colon cancer stemness, we analyzed NANOG protein expression in *FOSL1*-overexpressing DLD1 and HT-29 cells by western blot (Fig. 4a; Figure S6a). *FOSL1* expression resulted in an increment of NANOG in both cell lines. Moreover, overexpression of *FOSL1* enhanced the transcriptional activities of the *NANOG*-luc and *SOX2*-luc reporters in DLD1 cells but not of *LGR5*-luc (Fig. 4b). Quantitative reverse transcriptase–polymerase chain reaction (PCR) analysis confirmed the upregulation of endogenous *NANOG* gene expression, though not of *SOX2* and *LGR5*, in *FOSL1*-overexpressing DLD1 cells (Fig. 4c).

Clinical validation of these results was provided by the positive correlation between *FOSL1* (encoding for FRA1) and *NANOG* mRNA level in one CRC patient cohort from the GSE24551-GPL5175 [26] dataset (Figure S6b).

To evaluate whether *NANOG* is directly regulated by FRA1, we first analyzed in silico its promoter sequence using the JASPAR database (http://jaspar.genereg.net/) and predicted a FRA1/AP-1-binding site (FBE) 45–56 nucleotides upstream of the translational start site. Chromatin IP–quantitativePCR analysis confirmed that in DLD1 cells both wild-type FRA1 and FRA1-K116R, though not FRA1-K116Q, bind to the *NANOG* gene promoter through the FBE site (Fig. 4d). Similar results were obtained by *NANOG* gene luciferase reporter assays and by *NANOG* gene promoter DNA pull-down assays, respectively (Fig. 4e, f). Moreover, treatment with the HDAC6 inhibitor Tubastatin A significantly decreased *NANOG-*luc activity (Fig. 4g).

These results indicate that FRA1 upregulates *NANOG* gene transcription by directly interacting with its promoter. FRA1 deacetylation at K116 promotes its binding to the *NANOG* promoter.

### *NANOG* is a key downstream effector of IL-6/STAT3/FRA1-driven CSC properties

**Fig. 5:**
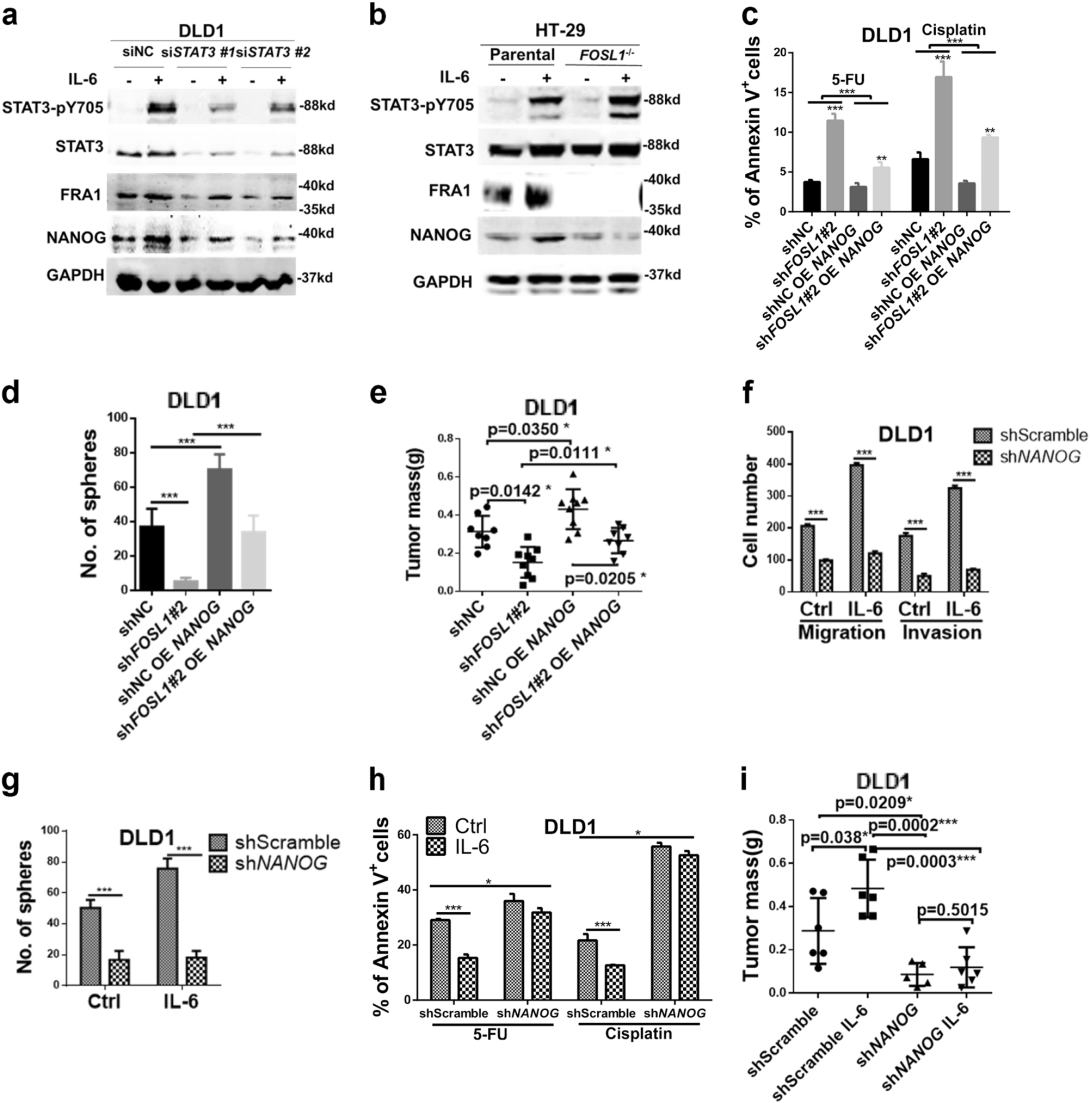
*NANOG* is a key downstream effector of interleukin (IL)-6/signal transducer and activator of transcription 3 (STAT3)/FRA1-driven CSC properties. **a** DLD1 cells were transfected with non-target control (siNC) or *STAT3* small interfering RNAs (siRNAs) and incubated with IL-6 for the indicated times. The protein levels of STAT3-pY705, STAT3, FRA1, NANOG, and GAPDH was examined by western blot analysis with the respective specific antibodies. **b** Parental (*FOSL1*^+/+^) and *FOSL1*^−/−^ HT-29 cells were cultured in the presence/absence of IL-6 for 24 h. The protein levels of STAT3-pY705, STAT3, FRA1, NANOG, and GAPDH was examined by western blot analysis with the respective specific antibodies. **c**–**e** Chemo-resistance (**c**), sphere (**d**), and xenograft (**e**) formation assays of DLD1 cells featuring different combinations of *FOSL1* knockdown, *NANOG* overexpression, and IL-6 stimulation (shNC = control for the siRNA-driven *FOSL1* knockdown). **f**, **g** Migration and invasion (**f**) and sphere formation (**g**) assays were performed with shScramble (control for the shRNA-driven *NANOG* knockdown) and sh*NANOG* DLD1 cells cultured in the presence/absence of IL-6. Results are shown as histograms showing quantitative values of the number of cells (or spheres) from triplicate experiments (mean ± SD). **h** shScramble and sh*NANOG* DLD1 cells were cultured in medium supplemented with the chemotherapeutic drugs 5-Fluorouracil and Cisplatin and in the presence/absence of IL-6 (50 ng/ml). The percentage of apoptotic (Annexin V^+^) cells indicate that sh*NANOG* DLD1 cells were characterized by increased sensitivity when compared to the shScramble cells. IL-6 enhances chemo-resistance in shNC DLD1 cells, an effect that is abrogated by the *NANOG* knockdown. **i** 5 × 10^5^ shScramble and sh*NANOG* DLD1 cells were cultured in the presence/absence of IL-6 for 5 days and then injected subcutaneously into the flanks of BALB/C nude mice. In the shScramble group, DLD1 cells treated with IL-6 formed larger tumors, an effect that was abolished upon *NANOG* knockdown. *p < 0.05, **p < 0.01, ***p < 0.001. Unpaired *t* test. Data are presented as mean ± SD

In view of the IL-6/STAT3-driven enhanced expression of both *FOSL1* and *NANOG* (Figs. 1a and 5a), and of the effects of FRA1 on *NANOG* transcriptional activity, we further investigated the role played by key TF as the downstream effector of the IL-6/STAT3/FRA1 signaling axis in CSC properties. We showed that siRNA-mediated *STAT3* knockdown markedly attenuated IL-6-induced FRA1 and NANOG expression levels in DLD1 cells (Fig. 5a). Notably, *FOSL1* gene knockout significantly blocked IL-6-induced *NANOG* expression without affecting *STAT3* activation in HT-29 cells (Fig. 5b). Thus *NANOG* is a downstream effector of FRA1 in response to IL-6/STAT3 stimulation.

Next, we overexpressed *NANOG* in DLD1 cells with reduced *FOSL1* expression (*FOSL1* knockdown) (Figure S6c). On its own, *FOSL1* knockdown greatly reduced chemoresistance to 5-FU and Cisplatin in DLD1 cells, and this inhibitory effect was accordingly rescued upon *NANOG* overexpression (Fig. 5c). Similar results pointing to an increment of CSCs upon *NANOG* expression were obtained by sphere-formation and mouse xenograft assays (Fig. 5d, e).

We then knocked down *NANOG* in DLD1 and HT-29 cells using a lentivirus-based shRNA approach (Figure S6d). The positive effects of IL-6 on migration, invasion, sphere-formation ability, and chemoresistance were significantly reduced by *NANOG* knockdown (Fig. 5f–h; Figure S6e-S6g). Consistent with the in vitro findings, *NANOG* knockdown abolished the tumor-enhancing effect of IL-6 (Fig. 5i). These data pointed at *NANOG* as an important downstream effector of the IL-6/STAT3/FRA1 signaling axis in the modulation of CSC properties.

### Increased FRA1 protein expression with low K116 acetylation correlate with IL-6 and NANOG levels and with poor prognosis among CRC patients

**Fig. 6:**
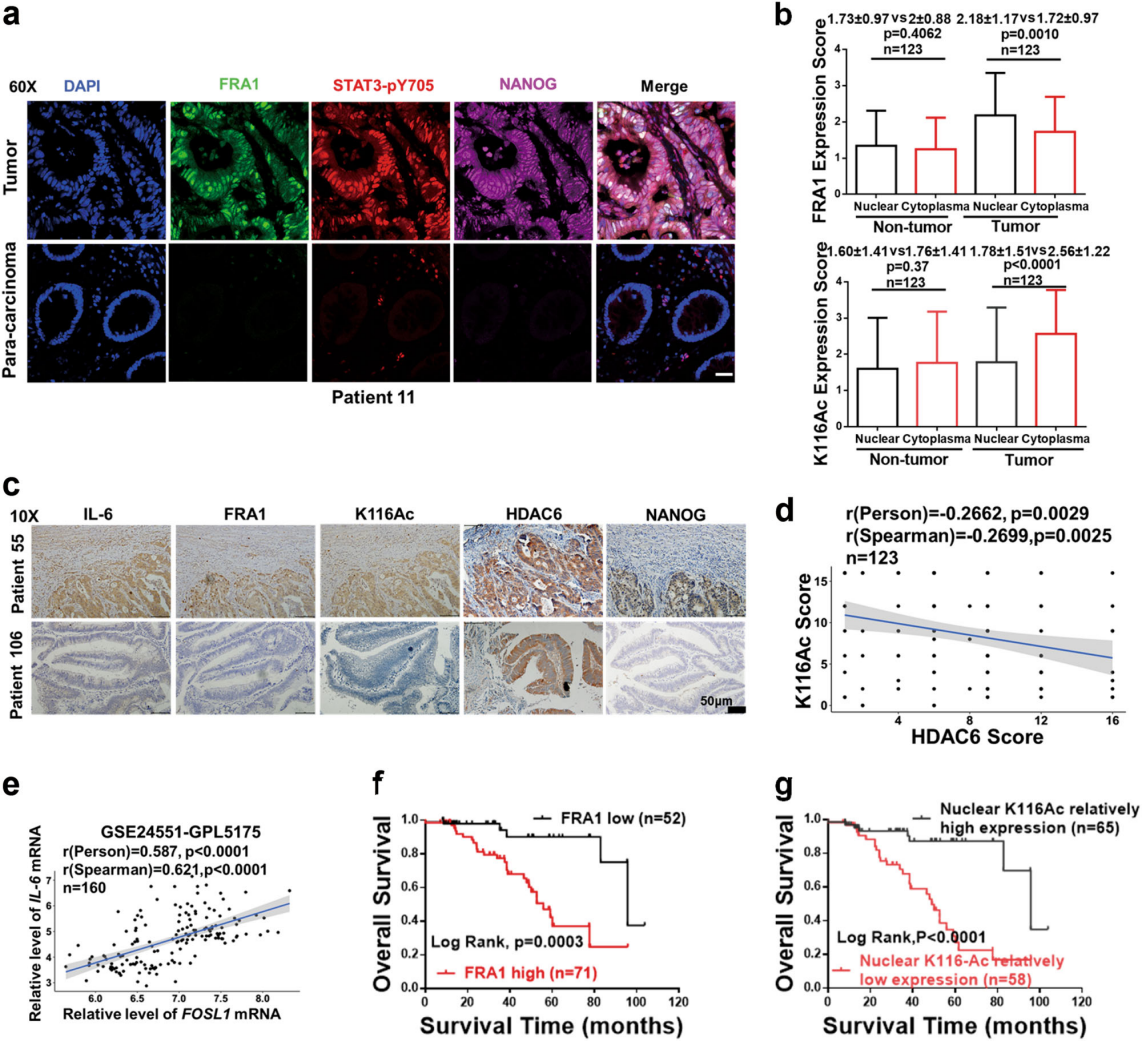
Increased FRA1 protein expression and low nuclear K116 acetylation correlate with increased interleukin (IL)-6 and NANOG levels and with poor prognosis among colorectal cancer (CRC) patients. **a** Representative immunofluorescent images of CRC tissues co-stained with antibodies directed against FRA1 (green), STAT3-pY705 (red), NANOG (carmine), and DAPI (blue). Scale bars represent 50 μm. **b** Quantification of immunohistochemically(IHC) determined FRA1 and K116Ac protein levels in nuclei and cytoplasm of cells located within CRC tissue and in normal tissue regions from CRC patients. **c** Representative IHC images of concurrent expression of IL-6, FRA1, K116Ac, HDAC6, and NANOG proteins in consecutive sections of CRC tissues from two different CRC patients. Scale bars represent 50 μm. **d** Correlation analysis of HDAC6 and K116Ac protein expression levels among CRC patients. **e** Correlation analysis of relative mRNA expression levels of *IL-6* and *FOSL1* from GSE24551-GPL5171 dataset [26]. **f**, **g** Overall patient survival negatively correlates with high FRA1 protein level (**e**) and with its low nuclear K116 acetylation (**f**). Log-rank (Mantel–Cox) test was here employed for survival analysis

To validate the correlation between malignant behavior of CRC and the expression levels of IL-6, K116Ac-FRA1, FRA1, and NANOG, a cohort of prospectively collected CRCs (*n* = 123) was employed. Immunofluorescence analysis showed increased expression and nuclear co-localization of FRA1, phosphorylated STAT3, and NANOG protein in the cancer samples when compared with adjacent (para-carcinoma) normal tissues (Fig. 6a; Figure S6h). Immunohistochemical (IHC) analysis with the anti-K116Ac antibody (whose specificity was previously validated; Figure S7a) showed that FRA1 was mostly identified in the nucleus of cancer cells while its K116-acetylated form mainly localized to the cytoplasm (Fig. 6b; Figure S7b), thus indicating that phosphorylated STAT3 can activate FRA1 both by transcriptional upregulation and deacetylation leading to increased NANOG expression levels.

Using consecutive tissue sections, IHC analysis further confirmed the positive correlation between the expression level of FRA1 and IL-6 secretion (Fig. 6c). Notably, cancer cells located in marginal areas adjacent to inflammatory regions displayed stronger FRA1 immunoreactivity as well as weaker K116Ac immunoreactivity when compared with those in the center of the cancer mass. The latter observations suggest a paracrine effect of inflammatory cells on FRA1 protein expression and K116 deacetylation on CRC cells. HDAC6 localizes to both the cytoplasm and the nucleus of cancer cells (Fig. 6c). Moreover, quantitative analysis of the IHC results indicated that HDAC6 levels inversely correlated with FRA1 acetylation at K116 in the CRC cohort (Fig. 6d). IL-6 and FRA1 protein expression was increased in tumor compared with non-tumor tissues and positively correlated in the CRC cohort (Figure S7c-S7e). These results were further validated with the GSE24551-GPL5175 dataset (Fig. 6e; Figure S7f and S7g).

Last, we evaluated the prognostic value of increased FRA1 combined with its decreased K116 acetylation in the prospective CRC cohort. CRC patients with high FRA1 and relatively low K116Ac expression are characterized by a significantly reduced overall survival when compared with those with carcinomas characterized by low FRA1 and relatively high K116Ac expression (Fig. 6f, g).

### Combined treatment of 5-FU with the HDAC6 inhibitor Tubastatin A synergistically inhibits CRC stem-like properties and malignant growth

**Fig. 7:**
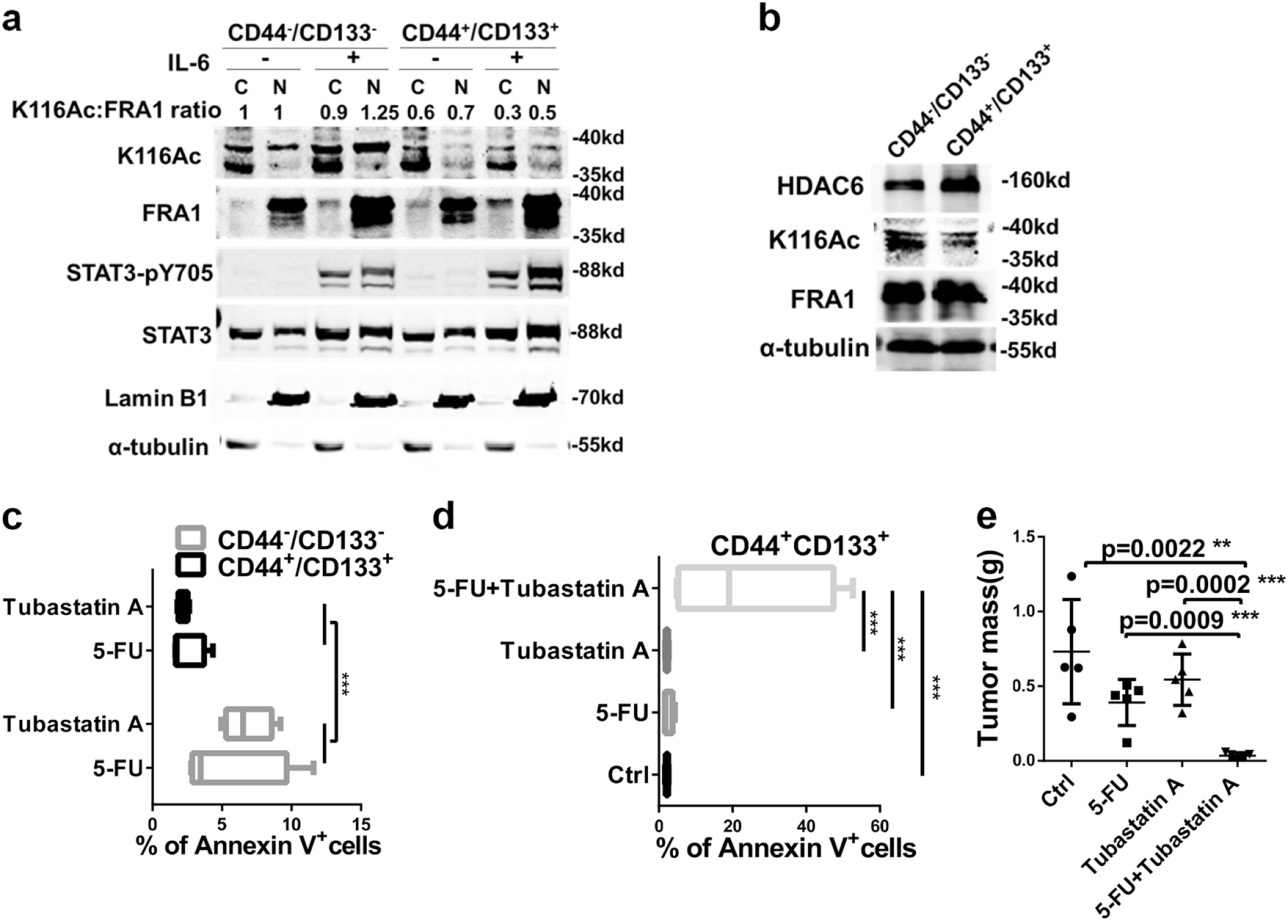
Combined treatment of 5-Fluorouracil (5-FU) with the HDAC6 inhibitor Tubastatin A synergistically inhibits colorectal cancer stem-like properties and malignant growth. **a** Western blot analysis of nuclear and cytoplasmic extracts from sorted CD44^−^/CD133^−^ and CD44^+^/CD133^+^ DLD1 cells (as in Figure S1i) cultured with control or interleukin-6 supplemented medium. K116 acetylation was detected with the K116Ac antibody and normalized against the FRA1 protein. **b** HDAC6 and FRA1 (total and K116Ac) western blot analysis of CD44^−^/CD133^−^ and CD44^+^/CD133^+^ DLD1 cells sorted by fluorescence-activated cell sorter. **c** Percentages of apoptotic (Annexin V^+^) cells in sorted CD44^−^/CD133^−^ and CD44^+^/CD133^+^ DLD1 cells when exposed to 5-FU or Tubastatin A. **d** Percentages of apoptotic cells in sorted CD44^+^/CD133^+^ DLD1 cells when exposed to dimethyl sulfoxide (Ctrl), 5-FU, Tubastatin A, and 5-FU plus Tubastatin A. **e** Effects of 5-FU, Tubastatin A, and of 5-FU with Tubastatin A administered to BALB/C nude mice on day 8 when tumor had a diameter of ~5 mm from CD44^+^/CD133^+^ DLD1 cells. Histogram showing the quantification of the tumor mass

Based on the results thus far, inhibition of the enzymatic machinery leading to FRA1-K116 deacetylation is expected to result in improved clinical outcomes especially in patients with increased IL-6 and FRA1 protein expression. To provide proof of principle for this concept, we first sorted and cultured CD44^−^/CD133^−^ and CD44^+^/CD133^+^ DLD1 cells in the presence/absence of IL-6 for 18 days to then harvest them and separate the cytoplasmic and nuclear protein fractions for western blot analysis. CD44^+^/CD133^+^ CSCs are characterized by lower K116 acetylation levels both in the nucleus and cytoplasm compared with their double-negative (CD44^−^/CD133^−^) counterparts (Fig. 7a). Upon IL-6 stimulation, FRA1-K116 deacetylation was observed in CD44^+^/CD133^+^ but not in CD44^−^/CD133^−^ cells. Likewise, we detected decreased K116Ac levels in CD44^+^/CD133^+^ compared with CD44^−^/CD133^−^ DLD1 cells (Fig. 7b). These results indicate that colorectal CSCs are relatively low in acetylated FRA1-K116 and that IL-6 can further lower the relative level of the modified protein in these stem-like cancer cells.

To investigate whether the combination of 5-FU and Tubastatin A is effective in targeting colon cancer stem cells and tumor growth, in vitro drug resistance and in vivo mouse xenograft assays were performed using sorted CD44^+^/CD133^+^ and CD44^−^/CD133^−^ DLD1 cells. We showed that, while CD44^+^/CD133^+^ cells were more resistant to 5-FU or Tubastatin A employed as single agents when compared with CD44^−^/CD133^−^ (Fig. 7c; Figure S8a), the combination of 5-FU with the HDAC6 inhibitor reversed their drug resistance phenotype (Fig. 7d; Figure S8b). Western blot analysis of the cells treated with the single and combined agents as in Fig. 7d showed that Tubastatin A alone or combined with 5-FU treatment resulted in increased expression of K116Ac and decreased expression of NANOG (Figure S8c). The 5-FU/Tubastatin A combination also had synergistic inhibitory effects on DLD1 cell proliferation (Figure S8d). Accordingly, the combined 5-FU/Tubastatin A treatment dramatically inhibited xenograft growth in vivo (Fig. 7e). Of note, the toxicity of the combined treatment was not higher than that of the single agents, as shown by the unchanged body weight among the different treated animals (Figure S8e). The results are also supported by the observed positive correlation between *HDAC6* and *NANOG* expression in the GSE24551-GPL5175 dataset (Figure S8f).

Overall, these results indicate that the combined treatment of the chemotherapeutic agent 5-FU with the HDAC6 inhibitor Tubastatin A may prove to be a potential novel curative approach for CRC patients, especially in those cases where the IL-6/STAT3/FRA1 signaling axis is activated.

## Discussion

In this study, we show that FRA1 is a key downstream effector of the IL-6–STAT3 signaling axis and that it accounts for increased colon cancer stemness and malignant behavior in the context of inflammation. We find that, upon IL-6/STAT3 signaling, HDAC6-driven deacetylation of a previously unrecognized K116 residue in FRA1 activates its transcriptional activity. Among the direct transcriptional targets of FRA1, *NANOG* plays a central role in modulating CRC stemness. Accordingly, increased FRA1 expression combined with low levels of K116 nuclear acetylation correlate with IL-6 secretion and with poor prognosis and overall survival among CRC patients. Notably, the combination of 5-FU with the HDAC6 inhibitor Tubastatin A may offer a potential novel therapeutic approach for colorectal CSCs.

Increasing evidence has shown that, upon inflammation, IL-6 activates STAT3 and exerts its predominant function in the regulation and maintenance of liver and colon CSCs [5, 27] However, the underlying molecular mechanisms and the IL-6/STAT3 downstream target genes that account for increased cancer stemness and malignancy are to date yet largely obscure. Previously, we reported that aberrant activation of STAT3, but not AKT or extracellular signal–regulated kinase (ERK), mediated the IL-6-induced upregulation of FRA1, required for EMT activation and increased CRC aggressiveness [3]. Here we employed human recombinant IL-6 to activate the IL-6/STAT3 pathway and found that it promotes colon cancer stemness in an FRA1-dependent manner. Consistent with our findings, the role of FRA1 in regulating CSC properties has also been reported in breast and liver cancer [17, 18]. In addition to IL-6, another inflammatory cytokine, IL-22, was also reported to promote pancreatic and CRC stemness via STAT3 activation [28, 29], which suggested that these two cytokines might coordinate with each other to modulate colorectal CSC properties.

The activation of FRA1 downstream of IL-6/STAT3 is known to mainly occur at the transcriptional level through pSTAT3-dependent upregulation of the *FOSL1* gene. However, more recent studies have revealed that protein modifications, e.g., phosphorylation of S265, T223, and T230 [17, 19], are critical for FRA1 stabilization and for the modulation of its downstream target genes. Also, FRA1 hetero-dimerizes with members of the JUN family, yet another level of downstream gene expression regulation [25, 30, 31].

Acetylation is known to regulate the functional activity of TFs associated with the acquisition of CSC properties [32, 33]. We show here that FRA1 is acetylated at a lysine residue, K116, located within its DBD. Unlike its C-terminal phosphorylation, K116 acetylation of FRA1 enhances its interaction with target DNA promoter sequences without affecting its stability and interaction with c-Jun. Ectopic expression of a de-acetylated (K116R) FRA1 allele results in a significant increase in self-renew and tumorigenic abilities. Although c-FOS is highly homologous to the DBD domain in FRA1, c-Fos acetylation at K148 (corresponding to K116 in FRA1) has not been reported. Moreover, knockdown of c-Fos had no evident impact on the IL-6-induced EMT changes, thus indicating that c-Fos and its potential acetylation is independent of IL-6/STAT3 pathway [3].

HDAC6 is a key histone deacetylase playing relevant functional roles in different cellular compartments. Although mainly localized in the cytoplasm, several lines of evidence suggest that HDAC6 can also reside in the nucleus and interact with nuclear proteins, including HDAC11; sumoylated p300; transcriptional corepressors such as ETO2 and L-CoR; and TFs such as NF-κB, Runx2, and P53 [34–36]. Here we show by western blot and IHC analysis that HDAC6 localizes to both the nucleus and cytoplasm of CRC cells and patient-derived tumor tissues. We identified HDAC6 as the enzyme responsible for FRA1 K116 deacetylation. FRA1 can physically interact with HDAC6 and this interaction is strengthened by IL-6/STAT3 signaling. Thus FRA1 regulation downstream of IL-6/STAT3 occurs at two distinct levels: through the binding of phosphorylated STAT3 to the *FOSL1* promoter leading to its increased transcription, and, at the posttranslational level, through deacetylation of the lysine 116 residue, located within the DBD and resulting in enhanced interaction with the promoter of its target genes.

Among the genes known to regulate CSC properties, we used JASPAR database (http://jaspar.genereg.net/) and predicted that only *NANOG*, *SOX2*, and *LGR5* encompass AP-1-binding sequences within their promoters, thus suggesting that they may represent downstream targets of the IL-6/STAT3/FRA1 signaling axis. As shown here, only *NANOG* does factually represent a direct FRA1 target gene. The physical interaction between FRA1 and *NANOG* occurs through a FRA1/AP-1-binding site (FBE) upstream of the translational start site. FRA1 deacetylation at Lys-116 enhances this interaction and further activates *NANOG* transcription.

It has been reported that CD24 regulates *NANOG* expression through STAT3 phosphorylation and that pSTAT3 (Y705) bound to the *NANOG* promoter drives tumor onset and self-renewal in the liver [12]. Moreover, it was shown that *NANOG* expression in CRC is regulated by extracellular insulin-like growth factor signaling via STAT3 phosphorylation [13]. Several studies have revealed the importance of NANOG as a CSC maintainer in liver cancer and CRC [10–12]. Our study showed that IL-6 treatment enhanced *FOSL1* and *NANOG* expression in a STAT3-dependent fashion. Of interest, *FOSL1* gene knockout significantly blocked IL-6-induced *NANOG* expression, while *NANOG* overexpression in *FOSL1* knockdown cells partially rescued the negative effects of *FOSL1* ablation on chemo-resistance, self-renewal, and tumorigenesis. Notably, *NANOG* knockdown significantly inhibited IL-6 induced in vitro ability of migration, invasion, chemo-resistance, and self-renew and in vivo ability of tumorigenesis. Hence, *NANOG* represents, presumably together with others [18], a bona fide downstream effector of FRA1, rather than STAT3, in the regulation of CSC properties downstream of IL-6. In human ESCs, transforming growth factor-β/Activin-responsive SMADs bind to the *NANOG* promoter and play an essential role in sustaining self-renewal [37]. We showed here that, in the context of inflammation and CRC, the regulation of the NANOG stem-like pathway was hijacked by the inflammatory IL-6/STAT3/FRA1 pathway. These results were validated in a large (*n* = 123) cohort of prospectively collected human colorectal carcinomas. We found that, while FRA1 was mainly localized in the nucleus of cancer cells, its acetylated form was mainly present in the cytoplasm, i.e., indicative of low K116Ac levels in its nuclear fraction. More importantly, high FRA1 protein expression and its relatively low K116 acetylation levels in the nucleus highly correlate with IL-6 secretion and with poor prognosis and overall survival among CRC patients. In summary, the results of western blots and IHC both showed that IL-6 deacetylates FRA1-K116 in the CRC cells.

The elucidation of the cellular and molecular mechanisms underlying the enhancement of colon cancer stemness downstream of the IL-6/STAT3/FRA1 signaling axis opens novel opportunities to develop alternative and possibly more effective therapeutic strategies. Small molecules inhibiting lysine acetylation (HDAC inhibitors) have been reported in the scientific literature, some of which with Food andDrug Administrationapproval, for the treatment of a broad spectrum of cancer-related pathologies. SAHA (also known as Vorinostat) was approved to treat cutaneous T cell lymphoma [20]. The antiepileptic drug valproic acid also inhibits HDACs and is currently being employed in several clinical trials for various indications [20]. Tubastatin A is a neuroprotective and specific inhibitor of HDAC6 [38]. HDAC6 maintains glioma stem cells (GSCs) through GLI1 signaling and its specific inhibition, when combined with X-ray irradiation, suppresses the tumorigenic capacity of GSCs in vivo [39].

5-FU represents one of the most common chemotherapeutic drugs in the treatment of CRC. However, its curative effects as a single agent are far from optimal. As observed in this study and supported by several previous reports, the stem-like CD44^+^/CD133^+^ colon cancer cells are chemo-resistant, a characteristic that is enhanced by IL-6 stimulation and the subsequent FRA1 deacetylation. Here we showed that the combination of 5-FU with the HDAC6 inhibitor Tubastatin A, designed to target both the more committed CRC cells (by 5-FU) and the small but chemoresistant CSC fraction (by Tubastatin A), dramatically inhibited tumor growth in vivo with no significant in vivo toxicity. Hence, the combined treatment of a chemotherapeutic agent with an HDAC6 inhibitor may offer an improved and novel curative approach for CRC patients, especially in those cases where the IL-6/STAT3/FRA1 signaling axis is activated.

In conclusion, upon inflammation and IL-6 secretion from the microenvironment, the STAT3 pathway becomes activated and promotes CRC stemness and malignancy through the transcriptional (promoter binding) and posttranslational (K116 deacetylation) upregulation of *FOSL1*/FRA1. HDAC6 is responsible for the deacetylation of the Lys-116 residue in FRA1. *NANOG* is a direct FRA1 target gene likely to play a central role in conferring increased stemness and malignancy to the cancer cell. Last, combinatorial treatment with a conventional cytotoxic agent (5-FU) and an HDAC6 inhibitor (Tubastatin A) may offer novel potential therapeutic options for CRC patients.

## Materials and methods

### Patient samples

A total of 123 human CRC samples were collected at the Second Affiliated Hospital of Zhejiang University School of Medicine after informed consent had been given by all patients. The clinicopathological characteristics of the clinical specimens are summarized in Supplementary Table S2.

### Statistical analysis

Data represent the mean ± SD of at least three independent experiments. Animal studies were performed blinded. No animals were excluded from study results. The animal experimental groups were blinded to the experimentalists during the experiment and data collection. BALB/C nude mice (male, 5 weeks of age) were selected for each group in a randomized fashion. The Log-rank (Mantel–Cox) test was used for survival analysis. The two-tailed *t* test was used to evaluate statistical differences in different groups. Pearson correlation and Spearman rank correlation tests were computed to assess the relativity of two groups. All analyses were performed using the GraphPad Prism6 software and R x64 3.4.4 statistical software. ^#,^*p < 0.05; ^##,^**p < 0.01; ^###,^***p < 0.001.

### Study approval

All animal studies were reviewed and approved by the Laboratory Animal Welfare Committee of Zhejiang University (approval number ZJU2015-282-01). The procedures related to human subjects were approved by the Medical Ethics Committee of Zhejiang University School of Medicine (approval number 2015-013).

See Online Supplementary Material and Methods for additional experimental procedures [40–49].

## Supplementary information

Revised supplementary marked up version

Revised supplementary clean version

supplementary figures
