# Supplementary material for: The inflammatory cytokine IL-6 induces FRA1 deacetylation promoting colorectal cancer stem-like properties
Source: Oncogene. 2019 Feb 25;38(25):4932–47. doi: 10.1038/s41388-019-0763-0 (PMC6756002; doi:10.1038/s41388-019-0763-0)
Supplement: Supplementary file 1 — Revised supplementary marked up version [file 41388_2019_763_MOESM1_ESM.pdf]

## Supplemental materials and methods

### Cell culture and transfection

Human CRC cell lines DLD1, HT-29 were obtained from the cell bank of Chinese Academy of Sciences (Shanghai, China), and the cell lines were authenticated by STR profling before distribution. These cell lines were maintained in RPMI 1640 supplemented with 10% fetal bovine serum (Gibco-Thermo Fisher Scientific). 293T was from American Type Culture Collect (ATCC) and cultured in DMEM supplemented with 10% fetal bovine serum (Gibco-Thermo Fisher Scientific). All cells were cultured at 37°C with 5% $CO_2$. Cells transfected with plasmids were performed using Lipofectamine 3000 (Invitrogen) or X-treme GENE HP DNA Transfection Reagent (Roche). Cells transfected with siRNAs were performed using Lipofectamine RNAi MAX (Invitrogen).

### Lentiviral Transduction

Several lentiviral vectors were used to transduce CRC cells and establish stable cell lines. The lentiviral overexpression vector pLVX-Puro and knockdown vector pLVX-shRNA1 were purchased from Clontech Laboratories. For knocking down *FOSL1* in CRC cells, lentiviral delivery of short hairpin RNAs (shRNA) targeting *FOSL1* (sh*FOSL1*#1 and sh*FOSL1*#2) and non-target control (shNC) were used. For luciferase labeling of DLD1 cells, a lentiviral vector pTSB-Luc-Puro was used. The stable cell lines were obtained by 1μg/mL puromycin selection.

### Antibodies and reagents

Antibodies specific to Flag (F1804; Sigma-Aldrich), Myc (sc-40; Santa Cruz Biotechnology Inc), NANOG (ab109250; ab90168, ab21624; Abcam), LaminB1 (ab133741; Abcam), CD44 (ab51037; Abcam), CD133 (70R-41557; Fitzgerald), FRA1 (ab124722; Abcam), (sc-28310; Santa Cruz Biotechnology), SOX2 (ab92494; Abcam), STAT3-pY705 (D3A7; Cell Signaling Technology), STAT3 (9139; Cell Signaling Technology), β-catenin (sc-7963; Santa Cruz Biotechnology Inc), HDAC6 (D2E5; Cell Signaling Technology), GAPDH (M1310-2; Hua An Biotechnology) α-tubulin (M15; Hua An Biotechnology), HDAC1 (sc-81598; Santa Cruz

Biotechnology), HDAC2 (sc-9959; Santa Cruz Biotechnology), HDAC3 (sc-376957; Santa Cruz Biotechnology), HDAC4 (sc-46672; Santa Cruz Biotechnology), HDAC5 (sc-133225; Santa Cruz Biotechnology), and Acetylated-lysine (9441S; Cell Signaling Technology) were purchased. To generate the acetyl-lysine 116-specific polyclonal antibody against FRA1 (K116Ac), synthetic peptide RRERNK(Ac)LAAAKC was coupled to keyhole limpet hemocyanin (KLH) as an antigen to immunize rabbits. The antibody was purified from the rabbit antisera by affinity-chromatography using the acetylated peptide, then the antibody against the counterpart non-acetylated peptide was removed by chromatography (Shanghai HuiOu Biotech). K116Ac was characterized by western-blotting under various conditions, such as peptide competition. Recombinant Human IL-6 was purchased from PEPROTECH. TPA, TSA, NAM, Tubastatin A, Romidepsin (FK228), Entinostat (MS-275) and Tocilizumab (TCZ) were purchased from Selleck.

**Sphere Formation Assay**

Single cells were seeded at 1000 cells per well in 24-well ultra-low-attachment plates (3473; Corning Life Sciences, Lowell, MA, USA). Cells were grown in sphere medium consisting of DMEM/F12 (Invitrogen) supplemented with B27 serum-free supplement (1:50; Invitrogen), 20ng/ml epidermal growth factor and 10ng/ml basic fibroblast growth factor (PEPROTECH) at 37°C in 5% $CO_2$。Supplemented medium was added to each well every two days to sustain supplement supply. After 1-2 weeks the developed spheres (>100 μm) were counted and analysed using ImageJ software, with their image captured under the light microscope.

**Invasion and migration assays**

The invasion assay was performed as described [3]. The migration assay was done with 24-well transwell (Corning). $4*10^5$ DLD1 and HT-29 cells were suspended in 300μl serum-free RPMI 1640 and loaded onto the upper compartment whereas complete medium with or without 50ng/ml IL-6 was applied to the lower compartment. After incubation for 72h, the cells that had migrated to the lower surface of the membrane were fixed with 2% PFA and stained with crystal violet. The air-dried membrane was viewed under a microscope and four random

fields were selected for cell counting.

**Chemo-resistance assay**

Chemo-resistance of cells to 5-FU, Cisplatin and Tubastatin A were carried out using Annexin V/ PI staining assay. Cells were stained in 1x Annexin V binding buffer, FITC-conjugated Annexin V and PI solution as provided by the Annexin V/ PI Staining Kit (MultiSciences Biotech) according to manufacturer's instructions. The analysis was determined by a Cytomic flow cytometer 500 (BECKMAN COULTER).

**Flow cytometry**

For flow cytometry, the antibodies used included FITC-conjugated CD44 (eBioscience) and PE-conjugated CD133 (eBioscience). Rat IgG2b Iso Control FITC and Mouse IgG1 K Iso Control PE (eBioscience) were used to serve as controls. Cells were incubated with the antibodies in PBS containing 2% FBS. The samples were analyzed or sorted on the Cytomic flow cytometer 500 (BECKMAN COULTER) and BD FACS S ORP ARIA II (BD Biosciences), respectively. The data were analyzed by CXP Software and BD FACS S ORP ARIA II Software. Sorted cells were seeded in 6-well plates with $1*10^5$ cells per well. Plates were maintained at 37°C in a humidified incubator with 5% $CO_2$ treated with or without IL-6.

**Immunohistochemistry and Immunofluorescence**

A total of 123 human CRC samples were collected at the Second Affiliated Hospital of Zhejiang University School of Medicine after informed consent had been given by all patients. The immunohistochemistry and the scoring for each slide were performed as described [3].

For staining human CRC tissues, the tissues were cut into frozen sections and fixed in ice-cold 4% paraformaldehyde (PFA). After dehydration in 30% sucrose, tissues were embedded with Optimum Cutting Temperature compound (O.C.T. Compound) into small silicone tubes. Frozen tissue was sectioned into 10 μm thick sections with a cryostat. Sections were blocked in PBT (0.3% Triton in 1X PBS) + 5% NGS (normal goat serum) + 0.1% NaN3 and then stained with primary antibodies. Following incubation, the sections were washed and stained with

fluorochrome-conjugated secondary antibodies and counterstained with DAPI. Fluorescence signal was then visualized using OLYMPUS IX83-FV3000-OSR (Olympus). The primary antibodies used included anti-FRA1 (Santa Cruz), anti-NANOG (Abcam), anti-STAT3-pY705 (CST). The second antibodies used included Alexa Fluor 488 Goat Anti-Mouse IgG, Alexa Fluor 555 Goat Anti-Rabbit IgG and Alexa Fluor 647 Goat Anti-Chicken IgG (Life Technologies).

**Quantitative real-time PCR analysis**

Total RNA was isolated from cells using RNAiso Plus (TaKaRa, Kyoto, Japan). Reverse transcription was performed with the PrimeScript RT reagent Kit (TaKaRa). Quantitative real-time PCR (qRT-PCR) was achieved using the CFX96 Real-Time System (BIO-RAD). Target gene expression was normalized to GAPDH levels in respective samples as an internal control, and the results are representative of at least three independent experiments.

**Primer sequences for quantitative real-time PCR analyses.**

| Gene | Forward Primer (5' -> 3') | Reverse Primer (5' -> 3') |
|---|---|---|
| *FOSL1* | AGTCAGGAGCTGCAGTGGATGGT | TCAGTTCCTTCCTCCGGTTCCTGC |
| *NANOG* | CCAACATCCTGAACCTCAGCTA | TCTGCGTCACACCATTGCTA |
| *POU5F1* | GCCGCTGGCTTATAGAAGGT | CTCTCCCCAGCTTGCTTTGA |
| *SOX2* | GAGAGTGTTTGCAAAAGGGGG | CCGCCGCCGATGATTGTTA |
| *GAPDH* | ATGGGGAAGGTGAAGGTCGGAGT | TGACAAGCTTCCCGTTCTCAGCC |

**Western blot analysis**

Proteins were separated by SDS-PAGE and transferred to nitrocellulose membrane (GE Whatman). The membrane was probed with primary antibodies followed by incubation with IRDye 800CW or IRDye 680-conjugated secondary antibodies and then visualized by the Odyssey Infrared Imaging System (LI-COR Biosciences, Lincoln, NE). FRA1 exhibits multiple bands ranging from 30-40 kDa on immunoblots mainly attributed to post-translational modifications and alternatively spliced variants of this protein [14,18,19] As the K116Ac

antibody recognizes lysine 116 acetylated FRA1, it is expected to detect multiple isoforms and post-translationally modified variants.

**Luciferase reporter assay**

The genomic regions surrounding the promoter of human *SOX2* and *LGR5* and were amplified by PCR and inserted into the pGL3 vector. The reporter constructs containing 2100bp of *SOX2* and *LGR5* promoter were generated by subsequent PCR-based cloning. p*NANOG*-luc was obtained from Addgene, FRA1-luc was used as described [3]. Cells were transfected the reporter construct with wild-type or mutant *FOSL1* expression vectors. The pRL-TK was co-transfected in each experiment as an internal control for transfection efficiency. In indicated experiments, at 12h post-transfection, cells were incubated with Tubastatin A for 12 hrs and the luciferase activities were measured using the Dual-Luciferase Reporter Assay System (Promega, Madison, WI) and a luminometer (LB 9507, Berthold, Bad Wildbad, Germany). The firefly luciferase activity was corrected by the corresponding Renilla luciferase activity and all experiments were carried out in triplicate.

**Immunoprecipitation**

293T and DLD1 cells were harvested with immunoprecipitation lysis buffer. Cell lysates were incubated overnight at 4°C with anti-Flag M2 magnetic beads (M882, Sigma-Aldrich) after removal of debris by centrifuging at 4°C, 13000 g for 15 min. The beads, which contained immunoprecipitate, were washed 3 times with lysis buffer and centrifuged at 400 g for 1 min between each wash. The beads were boiled and centrifuged at 4°C before loading on 10% SDS-PAGE gels and transferred onto nitrocellulose membrane (GE Whatman) for western blot analysis. The primary antibodies to Flag (F1804; Sigma-Aldrich), Myc (sc-40; Santa Cruz Biotechnology Inc), STAT3-pY705 (D3A7; Cell Signaling Technology), STAT3 (9139; Cell Signaling Technology), FRA1 (ab124722; Abcam), HDAC6 (D2E5; Cell Signaling Technology)

**Chromatin immunoprecipitation assay**

DLD-1 cells were transfected with wild-type Flag-*FOSL1* and indicated mutated derivatives

with Flag tag. Chromatin was immunoprecipitated using SimpleChip Kit according to manufacturer's instructions (9003; Cell Signaling Technology). The Chip-enriched DNA was then purified and analyzed by qPCR analysis using the following pair for *NANOG* promoter containing FBE site: 5'-GAACAAAAGTCAGCTTGTGTGG-3' (forward) and 5'-GGTTTCTTGAATGTTGGGTTTG-3'(reverse).

**Enzyme-linked immunosorbent assay (ELISA)**

For the determination of IL-6 concentration in the culture medium of DLD1 and HT-29 CRC cell lines, human IL-6 ELISA kit (430501; Biolegend) was used according to manufacturer's instruction.

**Cell Viability Assay**

The Viability of cells was carried out using CELL-Counting Kit-8 (CCK8) (DOJINDO) according to the manufacturer's instruction.

**DNA pull-down assay**

The DNA sequences covering from −404 to -1 of the human *NANOG* promoter and its mutants in predicated FRA1 binding site (FBE) site were amplified by PCR with the primers (one of which was labeled with biotin) and purified using a cycle purified kit (Omega). Wild-type, K116R and K116Q mutants of *FOSL1* were stably overexpressed in *FOSL1* previously knockdown DLD1 cells. A total of 100 μg nuclear proteins extracted from indicated knocking down and putting back stable DLD1 cell lines were obtained with buffer A (10mM HEPES, 1.5mM MgCl2, 10mM KCl, 0.5mM DTT, 0.05% NP40 (or 0.05% Igepal or Tergitol), pH7.9), and buffer B (5mM HEPES, 1.5mM MgCl2, 0.2mM EDTA, 0.5mM DTT, 26% glycerol (v/v), pH7.9). The biotinylated DNA probes were pre-incubated with M-280 streptavidin Dynabeads (Invitrogen) in binding and washing buffer (5mM Tris–HCl pH7.5, 500nM EDTA, 1M NaCl) according to the manufacturer's instructions. The beads were then added to the nuclear extracts and shaken for 4h at 4 C. After four washes with wash buffer (25mM HEPES pH7.5, 20% glycerol, 0.4% TritonX-100, 0.5mM EDTA, 150mM NaCl), protein loading buffer was

added to the precipitates, boiled for 5min, and separated on 10% SDS-PAGE. Proteins pulled down by the DNA probes were analyzed using western blot with Ku80 as a loading control.

**Metastases formation in nude mice**

Luciferase-labeled DLD1 cells ($3*10^5$/0.2 ml) were injected into the lateral tail vein of BALB/C nude mice. In weekly intervals, anesthetized mice were injected i.p. with D-luciferin (150 mg/kg) and imaged 10 min after injection using the IVIS Illumina System (Caliper Life Sciences). The acquisition time was 2 min. Eleven weeks after tail vein injection, mice were sacrificed and examined for lung metastases using H&E staining.

**Subcutaneous tumor growth in nude mice**

For subcutaneous (s.c.) injections, indicated cells were resuspended with 100 μl of 1×PBS and injected into each flank of BALB/C nude mice (male, 5 weeks of age). The mice were sacrificed three weeks later. Tumor tissues were harvested and tumor weight was measured.

**Combination therapy in mouse tumor model**

$CD44^+/CD133^+$ ($1*10^5$ cells/mouse) DLD1 cells were injected s.c. in the right flank of mice. Body weight and tumor volume were measured every other day. Mice were divided into 4 groups (n ¼ 5) using a stratified randomization strategy. Intraperitoneally (i.p.) injections of control (4% DMSO), Tubastatin A (0.5mg/kg/day), 5-FU (5mg/kg/day), or Tubastatin A (0.5mg/kg/day) plus 5-FU (5mg/kg/day) were initiated on day 8 when tumor had a diameter of~5 mm. On day 22, all mice were sacrificed and tumors weight was measured.

**Homology modeling and residue mutation**

The homology model of FRA1 (99-199aa) was constructed based on the crystal structure of c-Fos downloaded from the Protein Data Bank (PDB ID:1FOS) by EasyModeller v4.0 [40,41]. The sequence identity between FRA1 and c-Fos is 71.667%. The local geometries of the homology model were assessed by PROCHECK. The occupancy of residues (non-glycine and non-proline) in the most favorable regions in the Ramachandran plots for the homology model

is 100%, indicating the model is reasonably reliable. The FRA1 homology model was aligned to the c-Fos chain in the template (PDB ID:1FOS, RMSD = 3.84 Å), and then the c-Fos Chain in the crystal structure was replaced by the homology model to generate the whole complex using Pymol 2.0, which contains a chain of FRA1, a chain of c-Jun, and a DNA segment of 20bp oligonucleotides. The side chains of Arg112, Glu113 and Lys116 in FRA1 was found to overlap with DNA, and thus they were refined by *Prime* in Schrodinger 2017 [42]. Two mutated models of the initial complex were then generated by mutating Lys116 in FRA1 to Gln116 and Arg116, respectively. The two mutated FRA1 proteins were named as FRA1-K116Q and FRA1-K116R, and the three complexes were named as COM, COM-K116Q and COM-K116R.

**System preparation and MD simulations**

The three prepared structures were used as the initial structure for the conventional MD simulations. The topology and coordinate files of each system were generated by tleap in AMBER16. The ff14SB and OL15 force field was used [43]. Each system was solvated into a cubic TIP3P water box with 12 Å away from the surface of the complex. Then, an appropriate number of $Na^+$ ions were added to neutralize each system. The minimization, heating and equilibration processes were conducted by the *pmemd* program in AMBER16 [44]. In minimization, a restraint force of 10.0 kcal·mol$^{-1}$·Å$^{-2}$ was exerted on the FRA1, c-Jun and DNA atoms, and the solvent and ion molecules were optimized by 4000 cycles of steepest descent and 6000 cycles of conjugate gradient minimizations. Subsequently, all the atoms were optimized by 4000 cycles of steepest descent and 6000 cycles of conjugate gradient minimizations without any restraint. Each optimized system was heated from 0 to 300 K over a period of 0.2 ns with the solute atoms restrained by an elastic force of 10.0 kcal·mol$^{-1}$·Å$^{-2}$. Then, each system was equilibrated over 0.4 ns in the NPT ($T$ = 300 K and $P$ = 1 bar) ensemble. Finally, 100 ns MD simulations were conducted in the NPT ensemble. The temperature was controlled by the Langevin temperature equilibration scheme with a collision frequency of 2.0 ps$^{-1}$. The particle mesh Ewald (PME) algorithm was used to handle the long-range electrostatic interactions under periodic boundary condition and a cutoff of 8 Å was used for

the real-space interactions [44]. The SHAKE algorithm was used to constrain all covalent bonds involving hydrogen atoms and the time step was set to 2 fs.

**Trajectory analysis and MM/GBSA calculation**

Trajectory analysis was carried out with *cpptraj* module in Amber16. In order to monitor the stability of each system, the root mean square deviation (RMSD) of twenty residues in DNA combine region of FRA1 (Arg107 to Arg126) was calculated with their Cα atoms.

The binding free energy ($\Delta G_{bind}$) between protein (FRA1 and c-Jun) and DNA was calculated with the MM/GBSA methodology based on the snapshots from a single MD trajectory for each complex as single-trajectory protocol was adopted [45-47]. In MM/GBSA, the binding free energy was estimated according to the following equation [48].

$$\Delta G_{bind} = G_{complex} - G_{protein} - G_{DNA}$$

$$= \Delta H + \Delta G_{solvation} - T\Delta S$$

$$= \Delta E_{MM} + \Delta G_{GB} + \Delta G_{SA} - T\Delta S$$

Where $\Delta E_{MM}$ is the gas-phase interaction energy between protein and DNA, containing electrostatic and van der Waals interactions; $\Delta G_{GB}$ and $\Delta G_{SA}$ represent the polar and non-polar components of the solvation free energy, respectively; $T\Delta S$ represents the change of conformational entropy upon ligand binding, but this term is not considered here because of its expensive computational cost and low prediction accuracy. The polar solvation energy is estimated using modified GB model developed by Onufriev and coworkers (igb=2). The interior dielectric constant was set to 3 due to relatively strong electrostatic interaction between protein and DNA. The non-polar term of desolvation was calculated by solvent accessible surface area (SASA) with the LCPO model: $\Delta G_{SA} = 0.0072 \times \Delta SASA$. The snapshots extracted from last 50ns of each trajectory were used to calculate the energy terms.

The interactions between each residue of AP-1 and DNA were characterized by the MM/GBSA free energy decomposition [49]. Three terms constitute the binding interaction between residue and ligand: van der Waals contribution ($\Delta G_{vdw}$), electrostatic contribution ($\Delta G_{ele}$) and desolvation contribution ($\Delta G_{solvation}$). The non-polar term of desolvation ($\Delta G_{SA}$) was calculated by solvent accessible surface area (SASA) with the ICOSA method (GBSA=2). and the other

terms were calculated based on the same parameters used in the total free energy calculations.

**Supplemental Figures and Legends**

**Figure S1. IL-6 promotes colon cancer stemness.**

**(a)** ELISA analysis of IL6 secretion in the culture medium from wild type, *FOSL1*-knockdown, and -overexpressing HT-29 and DLD1 cells.

**(b-c)** Migration and invasion **(b)**, and sphere formation **(c)** assays of DLD1 and HT-29 cells cultured in the presence/absence of IL-6. Results are shown as representative images and as histograms of the cell or sphere numbers in triplicate (mean ± SD). Scale bars represent 50 μm.

**(d)** DLD1 and HT-29 cells were treated with the chemotherapeutic drug 5-FU or Cisplatin in the presence/absence of 50 ng/ml of IL-6. The percentage of Annexin V-positive cells is a measure of the chemoresistance induced by IL-6 in CRC cells. Upper panel: representative Annexin V-FITC/PI FACS plots; lower panel: histograms of the quantification of the FACS results.

**(e)** Representative images of *in vivo* luciferase activity (upper panel), H&E staining and Ki67 IHC analysis (lower panel) of pulmonary metastatic foci obtained by tail-vein injection of DLD1 cells cultured in the presence/absence of IL-6 for 5 days. Scare bar represent 200 μm.

**(f)** Western blot analysis of DLD1 cells cultured in the presence/absence of IL-6 (50 ng/ml), TCZ (5μg/ml) and si*STAT3*. Protein levels of STAT3-pY705, STAT3, FRA1, and α-tubulin were examined.

**(g)** Sphere formation assays were performed with DLD1 cells cultured in the presence/absence of IL-6 (50 ng/ml), TCZ (5μg/ml) and si*STAT3*. Results are shown as representative images and as histograms of the sphere numbers in triplicate (mean ± SD). Scale bars represent 50 μm.

**(h)** DLD1 and HT-29 cells were cultured in the presence of IL-6 for 7 days and the $CD44^+/CD133^+$ subpopulation was assessed by FACS.

**(i-j)** Sphere **(i)** and xenograft **(j)** formation assays were implemented on sorted $CD44^-/CD133^-$

and CD44$^+$/CD133$^+$ DLD1 cells cultured in the presence/absence of IL-6. CD44$^+$/CD133$^+$ cells formed larger sphere and tumors when compared with CD44$^-$/CD133$^-$ cells. IL-6 treatment enhances these features in both subpopulations. Scale bars represent 50 μm.

**(k)** CD44$^+$/CD133$^+$ and CD44$^-$/CD133$^-$ DLD1 cells were sorted by FACS and cultured in the presence/absence of IL-6 for the indicated times. These cultures were monitored at the indicated time points by FACS analysis. Representative FACS plots are depicted.

*p<0.05, **p<0.01, ***p<0.001. Unpaired t-test. Data are presented as mean ± SD.

**Figure S2. IL-6 promotes colon cancer stemness in a FRA1-dependent manner.**

**(a)** Western blot analysis of *FOSL1* overexpression in DLD1 and HT-29 cells.

**(b)** Migration and invasion assays were performed with empty vector (EV) and *FOSL1* overexpressing DLD1 and HT-29 cells. Results are shown as representative images (upper panel) and as histograms of cell numbers in triplicate (mean ± SD; lower panel). Scale bars represent 50 μm.

**(c)** Sphere formation assays were performed with empty vector (EV) and *FOSL1* overexpressing DLD1 cells. Results are shown as representative images (left panel) and as histograms of sphere numbers in triplicate (mean ± SD; right panel). Scale bars represent 50 μm.

**(d)** $5*10^5$ EV and *FOSL1* overexpressing DLD1 cells were cultured in the presence/absence of IL-6 for 5 days to then be subcutaneously injected into the flanks of BALB/C nude mice. DLD1 cells overexpressing *FOSL1* increased tumor mass when compared with the empty vector (EV) group. As expected, IL-6 stimulation increased tumor mass in EV group.

**(e)** FRA1 western blot analysis of *FOSL1* knockdown in DLD1 and HT-29 cells.

**(f)** Migration and invasion assays were performed with non-target control (shNC), sh*FOSL1*#1 and sh*FOSL1*#2 HT-29 cells cultured in the presence/absence of IL-6. Results are shown both as representative images (upper panel) and histograms (lower panel) showing quantitative values of the number of cells from triplicate experiments (mean ± SD). Scale bars represent 50 μm.

**(g)** Sphere formation assays were performed with non-target control (shNC), sh*FOSL1*#1 and

sh*FOSL1*#2 HT-29 cells cultured in the presence/absence of IL-6. Results are shown both as representative images (left panel) and histograms (right panel) showing quantitative values of the number of spheres from triplicate experiments (mean ± SD). Scale bars represent 50μm.

**(h)** shNC and *FOSL1* knock-down (sh*FOSL1*#1 and sh*FOSL1*#2) HT-29 cells were cultured in medium supplemented with the chemotherapeutic drugs 5-FU and Cisplatin and in the presence/absence of IL-6 (50 ng/ml). The percentage of apoptotic (Annexin $V^+$) cells indicate that both sh*FOSL1*#1 and sh*FOSL1*#2 are characterized by increased sensitivity when compared to the shNC control cells.

*p<0.05, **p<0.01, ***p<0.001. Unpaired t-test. Data are presented as mean ± SD.

**Figure S3. FRA1 is acetylated at lysine 116 but had no influence on its stability and interaction with C-Jun.**

**(a)** 293T cells were transfected with the indicated plasmids and IP-Flag was performed. FRA1 acetylation was analyzed by western blot with pan acetyl-lysine antibody (Ace-Lys).

**(b)** 293T cells were transfected with the indicated plasmids and IP-Myc was performed. FRA1 acetylation was analyzed by western blot with Ace-Lys.

**(c)** DLD1 cells were transfected with CREB-binding protein (CBP) and *FOSL1*-2HA and IP-HA implemented. FRA1 protein expression and acetylation were analyzed by western blot with anti-HA and Ace-Lys antibodies. Input and immunoprecipitation reactions were separated by SDS-PAGE and Coomassie stained followed by gel band excision and analysis by mass spectrometry.

**(d)** Sequence alignment of FRA1 and c-Fos around the K116Ac target region.

**(e)** The Flag-EV, Flag-*c-FOS* and Flag-*FOSL1* construct was transfected into DLD1 cells, and immunoprecipitation with antibodies directed against Flag. FRA1 and c-Fos acetylation was analyzed by western blot with the K116Ac-specific antibody.

**(f)** Root mean square deviation (RMSD) of the twenty residues in the DNA-binding domain of FRA1 (Arg107 to Arg126) during the 100 ns of MD simulations.

**(g)** The residue-residue interaction spectra of the DNA binding to FRA1 (green line), FRA1-K116Q (red line) and FRA1-K116R (blue line). (left panel) The total energy contribution

of each residue, (middle panel) the non-polar energy ($\Delta E_{vdw}+\Delta G_{SA}$) contribution of each residue, (right panel) the polar energy ($\Delta E_{ele}+\Delta G_{GB}$) contribution of each residue.

**Figure S4. Acetylation at Lysine 116 of FRA1 had no influence on its stability and interaction with C-Jun.**

**(a)** 293T cells were transfected with wild type and mutant *FOSL1* followed by incubation with cycloheximide (CHX, an inhibitor of protein synthesis) for increasing periods of time. FRA1 protein level was analyzed by western blot with a Flag-antibody. △3 deletion mutant construct was employed as a positive control.

**(b)** 293T cells expressing ectopic wild type and mutant *FOSL1* were co-transfected with *Myc-c-Jun*. After IP-Flag, *FOSL1* and *c-Jun* expression was analyzed by western blot with anti-Flag and anti-Myc antibody. Dimerization-defective (L154A/L161A) mutant construct was employed as a positive control.

**(c)** DLD1 cells were transfected with the indicated expression constructs. Specificity of the newly generated antibody directed against K116-acetylated FRA1 was assessed by western blot with K116Ac, K116Ac plus K116Ac acetylated peptide and Flag.

**(d)** 293T cells were transfected with wild type and mutant *FOSL1* expression constructs. After IP-Flag, K116-acetylated FRA1 was analyzed by western blot with the K116Ac antibody.

**Figure S5. HDAC6 deacetylates FRA1 and underlies its transcriptional activation downstream of IL-6/STAT3.**

**(a)** Western blot analysis of endogenous HDAC1-6 expression level in DLD1, HT-29 and 293T cells.

**(b)** Western blot analysis of K116Ac and FRA1 levels in DLD1 cells treated with FK228 and MS-275 for 24 hrs.

**(c)** Cytoplasmic and nuclear protein fractions were separated for western blot analysis using the indicated antibodies in DLD1 and HT-29 cells.

**Figure S6. *NANOG* is a key downstream effector of IL-6/STAT3/FRA1–driven CSCs**

**properties.**

**(a)** Western blot analysis of *FOSL1* and *NANOG* expression in HT-29 cells stably transfected with the *FOSL1* expression construct.

**(b)** Correlation analysis of the relative *NANOG* and *FOSL1* mRNA levels in GSE24551-GPL5171datasets [26].

**(c)** Western blot analysis of *FOSL1* and *NANOG* expression in *FOSL1* knockdown DLD1 cells. shNC: non-target control.

**(d)** Western blot validation of *NANOG* gene knockdown in DLD1 and HT-29 cells.

**(e-f)** Migration and invasion **(e)**, and sphere formation **(f)** assays were performed with shScramble（control for the shRNA-driven *NANOG* knockdown), sh*NANOG* HT-29 cells cultured in the presence/absence of IL-6. Results are shown both as representative images (upper panel) and histograms (lower panel) showing quantitative values of the number of cells (or spheres) from triplicate experiments (mean ± SD). Scale bars represent 50μm.

**(g)** shScramble and sh*NANOG* HT-29 cells were cultured in medium supplemented with the chemotherapeutic drugs 5-FU and Cisplatin and in the presence/absence of IL-6 (50 ng/ml). The percentage of apoptotic (Annexin $V^{+}$) cells indicate that sh*NANOG* HT-29 cells were characterized by increased sensitivity when compared to the shScramble cells. IL-6 enhances chemo-resistance in shNC DLD1 cells, an effect that is abrogated by the *NANOG* knockdown.

**(h)** Representative IF images of CRC tissues co-stained with FRA1 (green), STAT3-pY705 (red), NANOG (carmine) and DAPI (blue). Scale bars represent 50 μm.

**Figure S7. Increased *FOSL1* expression with low K116 acetylation correlate with IL-6 and NANOG levels and with poor prognosis among CRC patients.**

**(a)** The specificity of the antibody raised against K116-acetylated FRA1 was determined by IHC after incubation of the antibody with the originally employed acetylated peptide. Representative images of concurrent expression of *FOSL1* and K116Ac in consecutive sections of CRC tissues was shown.

**(b)** Representative IHC images of consecutive sections of CRC tissues stained with the anti-FRA1 and anti-K116Ac antibodies. Scale bars represent 50 μm.

**(c)** Quantification of IHC-determined IL-6 protein level in paired colorectal cancer and matched normal tissue samples from CRC patients.

**(d)** Quantification of IHC-determined FRA1 protein level in paired colorectal cancer and matched normal tissue samples from CRC patients.

**(e)** Correlation analysis of protein expression levels of IL-6 and FRA1 from CRC patients.

**(f)** Relative mRNA expression of *IL-6* in 13 paired colorectal cancer and matched normal tissue samples from the GSE24551-GPL5171 dataset [26].

**(g)** Relative mRNA expression of *FOSL1* in 13 paired colorectal cancer and matched normal tissue samples from the GSE24551-GPL5171 dataset.

**Figure S8. Combined treatment of 5-FU with the HDAC6 inhibitor Tubastatin A synergistically inhibits CRC stem-like properties.**

**(a)** Representative FACS plots of the apoptotic (Annexin $V^+$) cells in sorted $CD44^-/CD133^-$ and $CD44^+/CD133^+$ DLD1 cells when exposed to 5-FU or Tubastatin A.

**(b)** Representative FACS plots of the apoptotic (Annexin $V^+$) cells in sorted $CD44^+/CD133^+$ DLD1 cells when exposed to DMSO (Ctrl), 5-FU, Tubastatin A, and 5-FU plus Tubastatin A.

**(c)** Western blot analysis of DLD1 cells obtained from Figure.S7b.

**(d)** DLD1 cells were seeded in 96 well plates and further treated with the indicated concentrations of 5-FU, Tubastatin A, and 5-FU plus Tubastatin A, respectively. Cell viability was performed using Cell counting kit 8.

**(e)** *In vivo* toxicity assay. For each treatment, the body weight of each individual animals was measured every other day. The means ± SD of each group at different days after treatment are shown.

**(f)** Correlation analysis of relative mRNA expression levels of *HDAC6* and *NANOG* from the GSE24551-GPL5171 dataset [26].

**Table S1. The MM/GBSA predicted binding free energies and the individual energy components for three systems (kcal mol-1).**

| System | Polar contributions | | Nonpolar contributions | | |
|---|---|---|---|---|---|
| | $\Delta E_{ele}$ | $\Delta G_{GB}$ | $\Delta E_{vdw}$ | $\Delta G_{SA}$ | $\Delta G_{bind}$ |
| COM | -4596.66±2.86 | 4551.14±2.70 | -123.67±0.40 | -20.81±0.05 | -189.97±0.45 |
| COM-K116Q | -4402.07±2.64 | 4354.08±2.50 | -119.24±0.41 | -20.61±0.05 | -187.81±0.47 |
| COM-K116R | -4658.45±4.13 | 4598.16±3.97 | -113.20±0.44 | -20.76±0.05 | -194.23±0.53 |

**Table S2. Clinico-pathologic parameters of colorectal cancer patients.**

| Characteristics | Number (%) |
|---|---|
| Gender | |
| Male | 78 (63) |
| Female | 45 (37) |
| Age | |
| Mean (Range) | 65 (18-98) |
| <65 | 40 (33) |
| ≥65 | 83(67) |
| Tumor location | |
| Colon | 78 (63) |
| Rectum | 45 (37) |
| Tumor size | |
| <5cm | 46 (37) |
| ≥5cm | 77 (63) |
| Depth of invasion | |
| T1 | 56(46) |
| T2 | 39 (32) |
| T3 | 16 (13) |
| T4 | 12 (9) |
| Lymph node metastasis | |
| N0 | 49 (40) |
| N1 | 35 (28) |
| N2 | 39 (32) |
| Distant metastasis | |

| | |
|---|---|
| M0 | 108 (88) |
| M1 | 15 (12) |
| TNM stage | |
| I | 45 (37) |
| II | 31 (25) |
| III | 37 (30) |
| IV | 10 (8) |
