# Supplementary material for: The inflammatory cytokine IL-6 induces FRA1 deacetylation promoting colorectal cancer stem-like properties
Source: Oncogene. 2019 Feb 25;38(25):4932–47. doi: 10.1038/s41388-019-0763-0 (PMC6756002; doi:10.1038/s41388-019-0763-0)

Figure S1. IL-6 promotes colon cancer stemness.

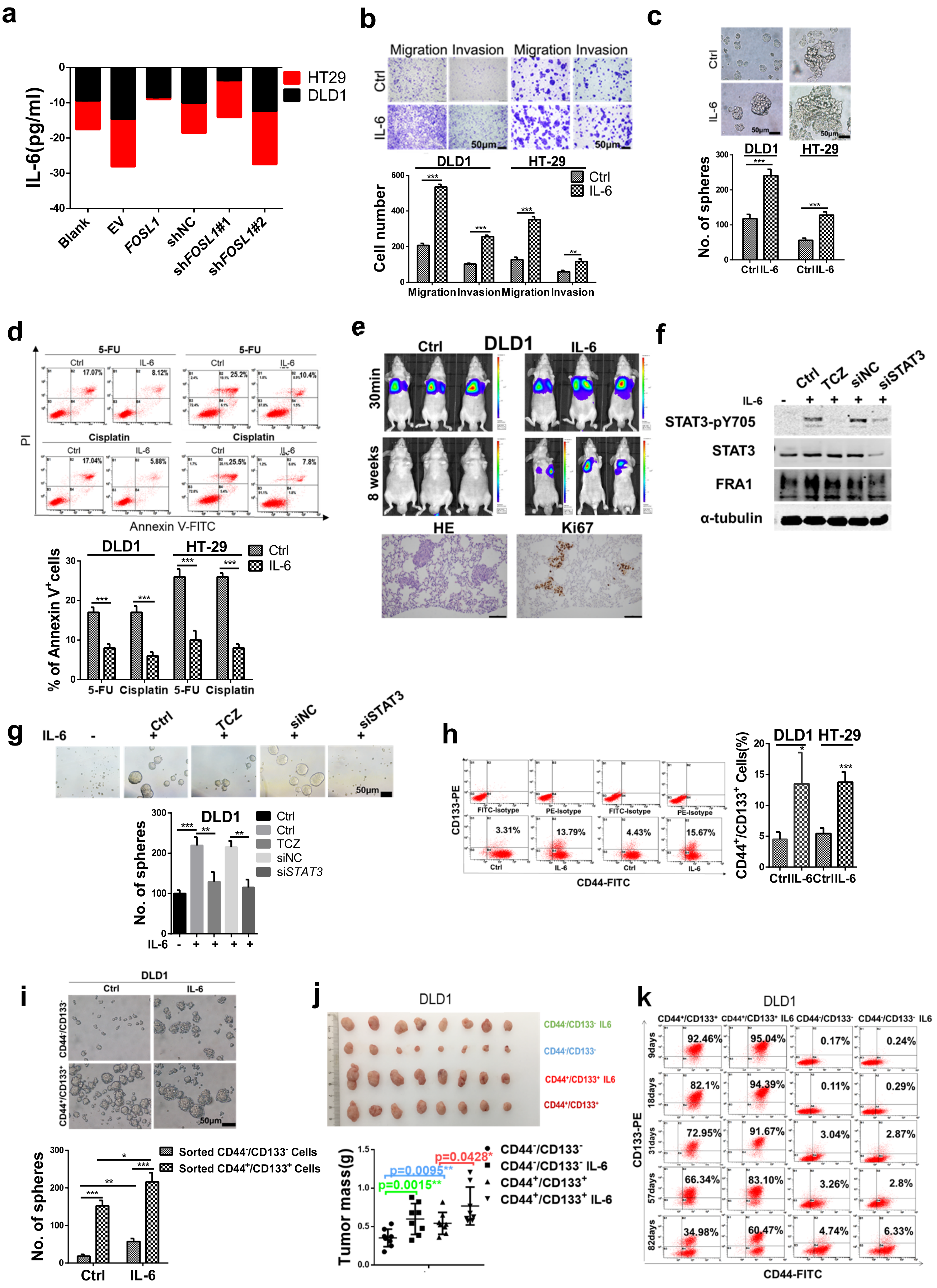

Figure S2 IL-6 promotes colon cancer stemness in a FRA1-dependent manner.

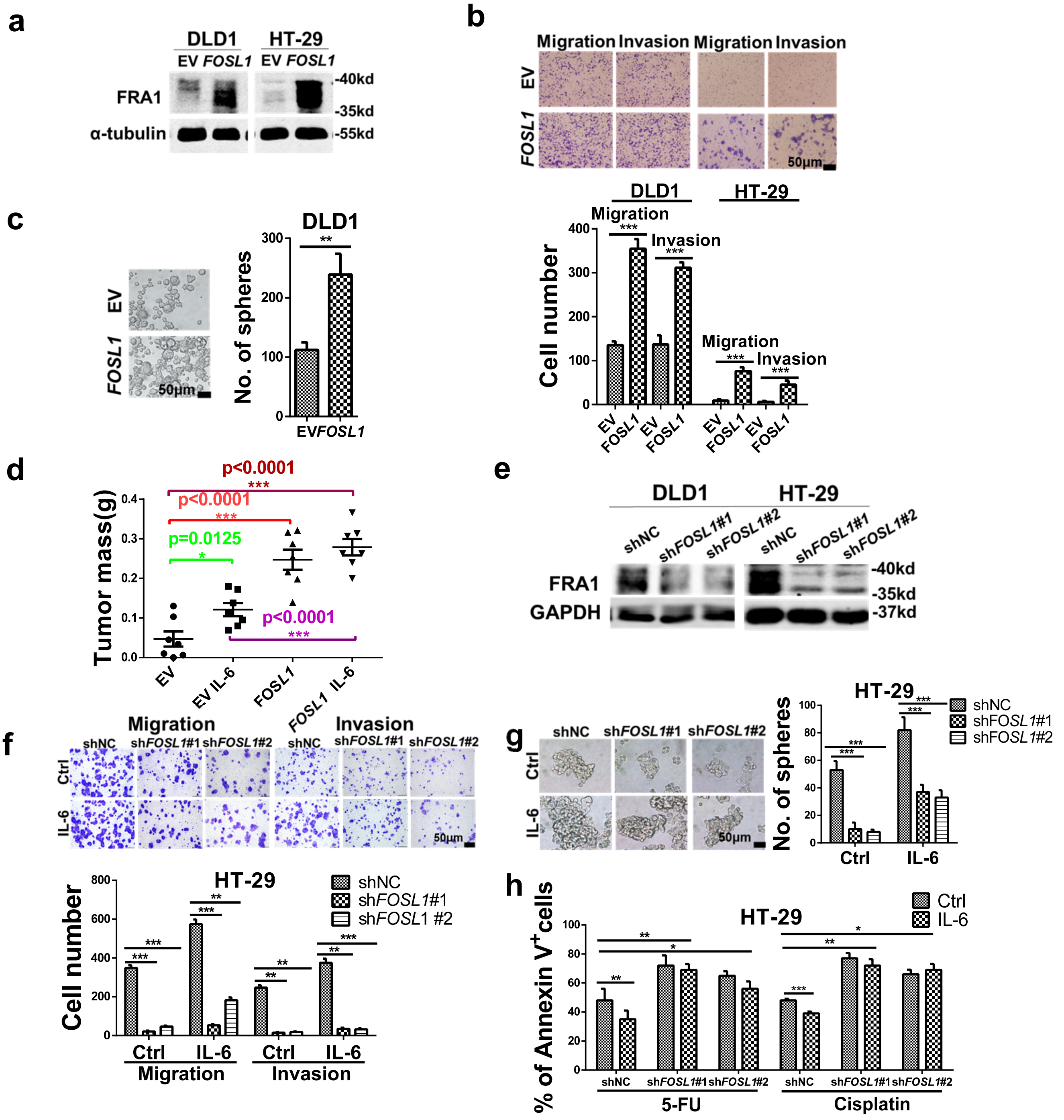

Figure S3. FRA1 is acetylated at lysine 116.

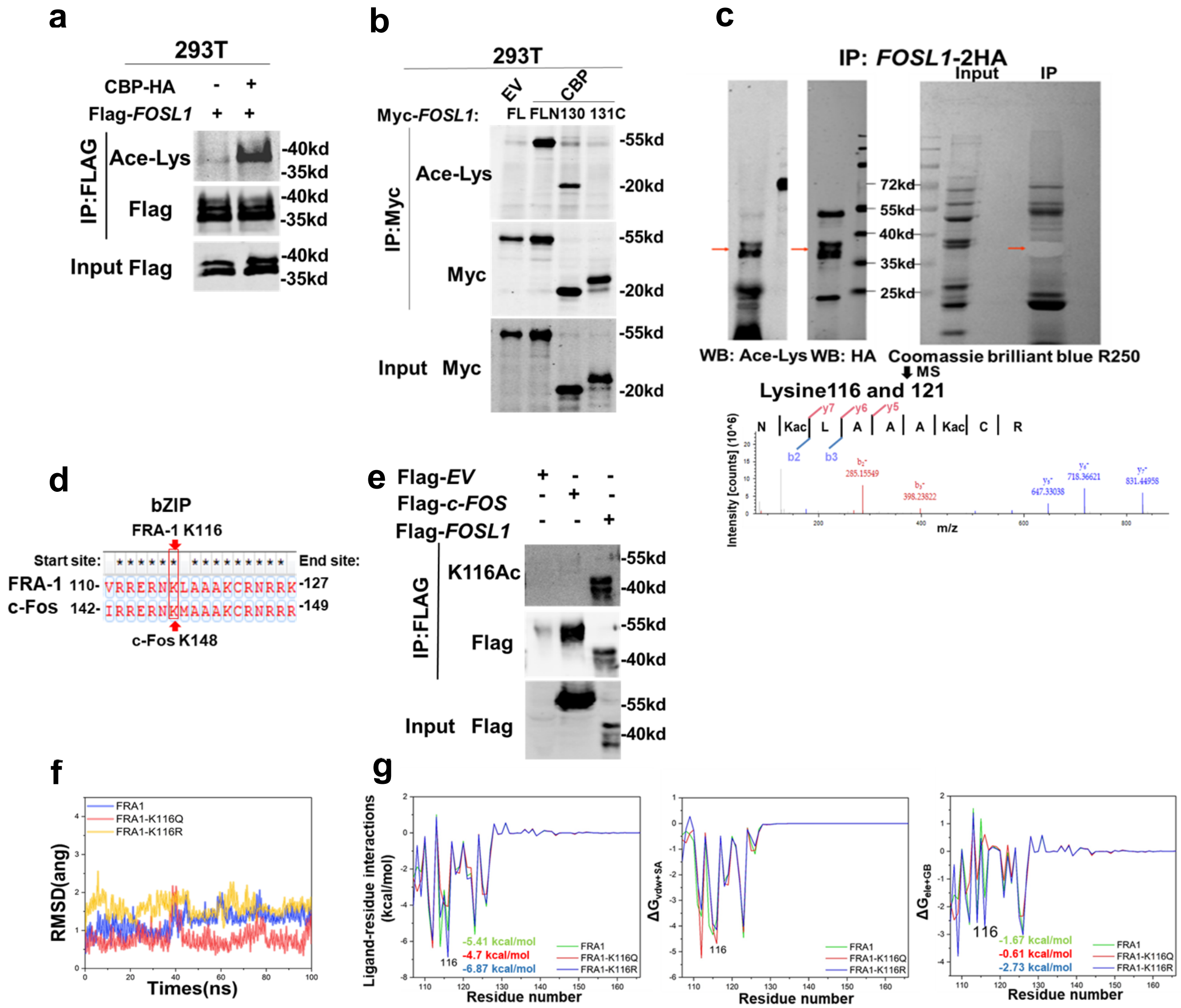

Figure S4. Acetylation at Lysine 116 of FRA1 had no influence on its stability and interaction with C-Jun.

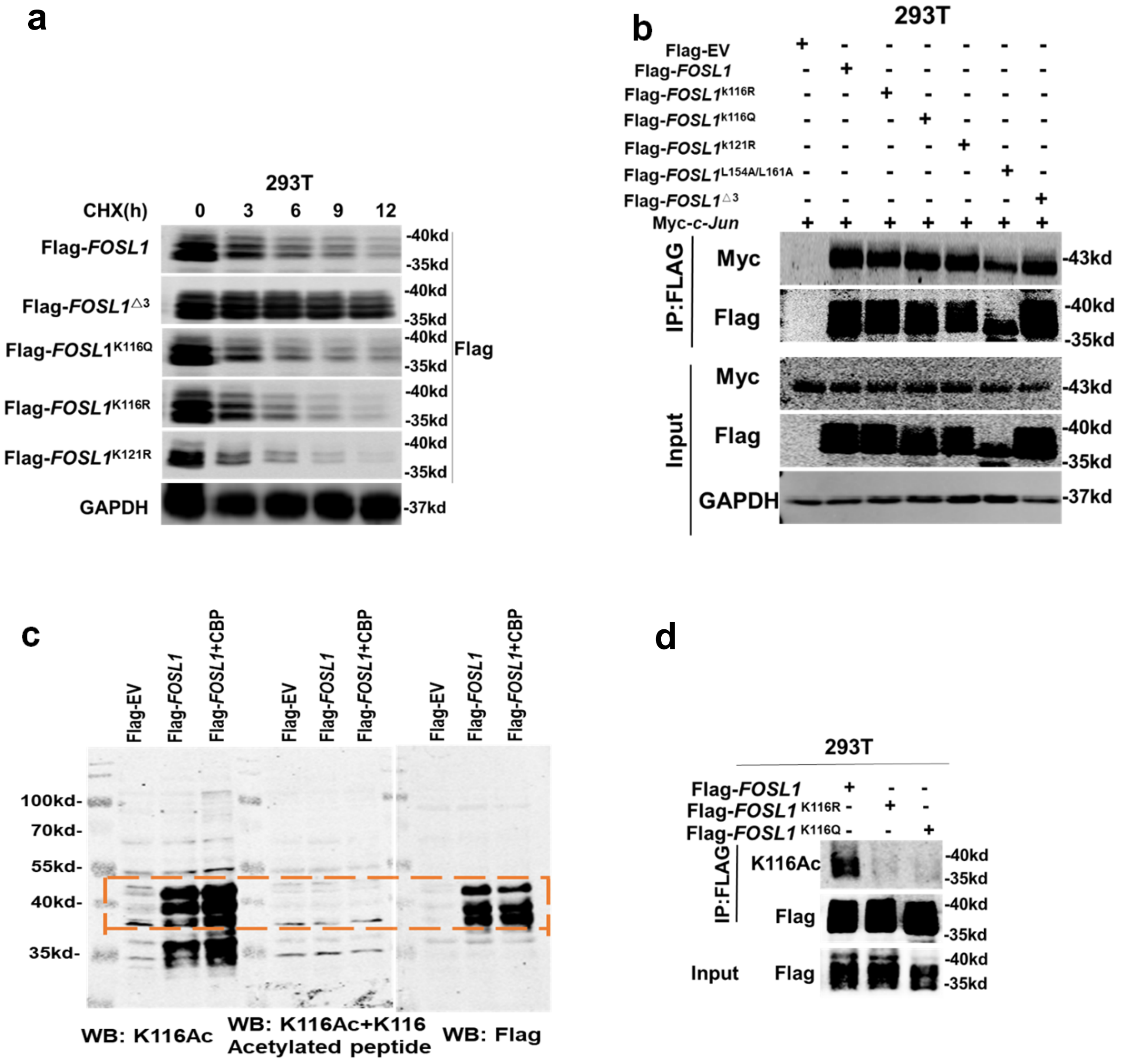

Figure S5. HDAC6 deacetylates FRA1 and underlies its transcriptional activation downstream of IL-6/STAT3.

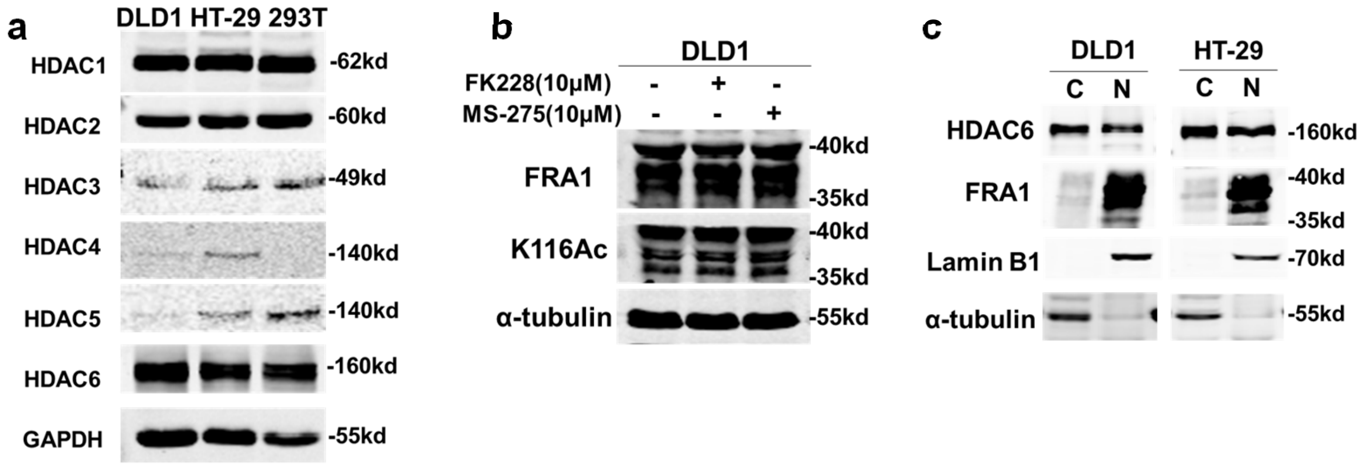

Figure S6. *NANOG* is a key downstream effector of IL-6/STAT3/FRA1–driven CSCs properties.

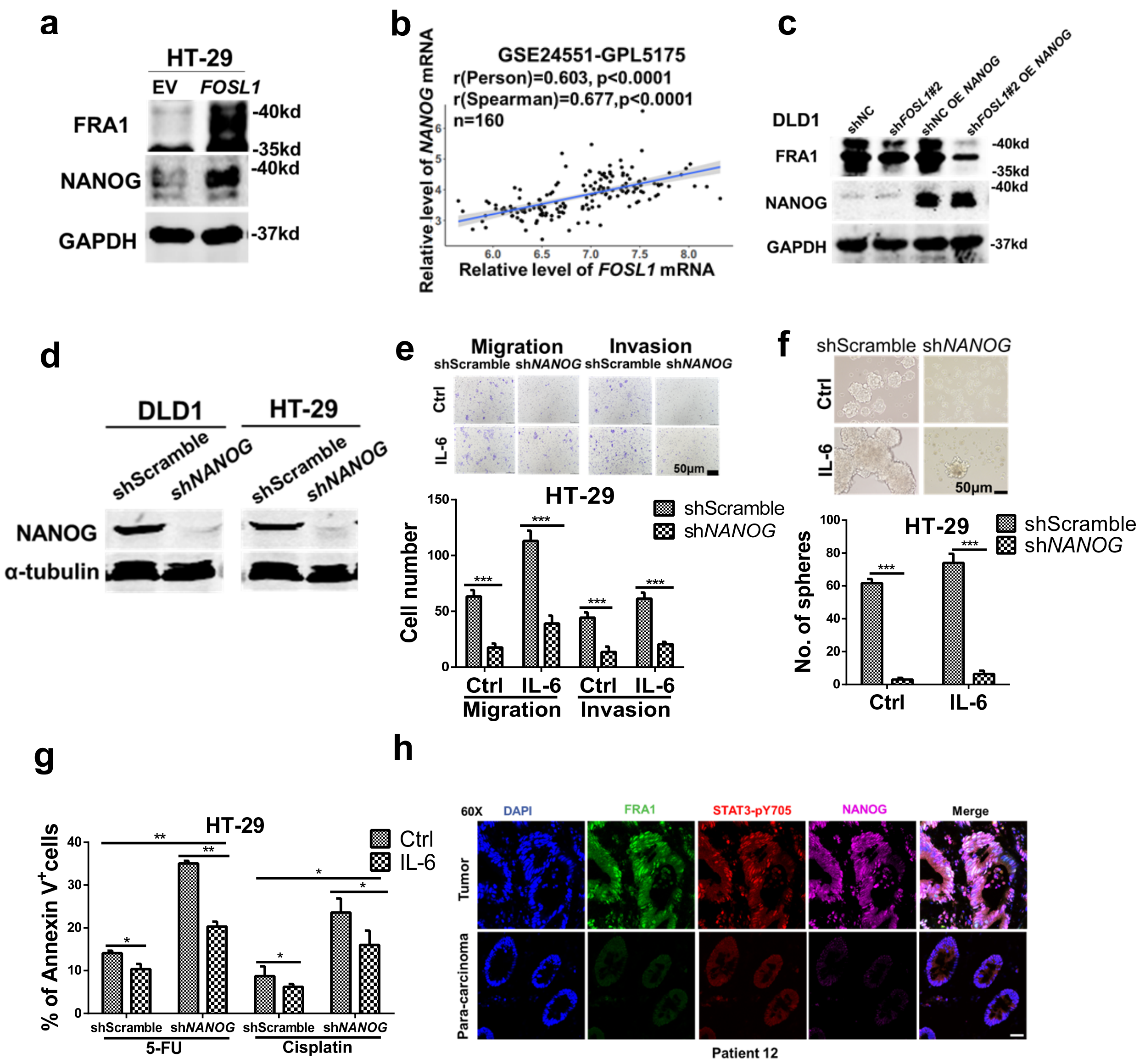

Figure S7. Increased FRA1 protein expression with low nuclear K116 acetylation correlate with IL-6 and NANOG levels and with poor prognosis among CRC patients.

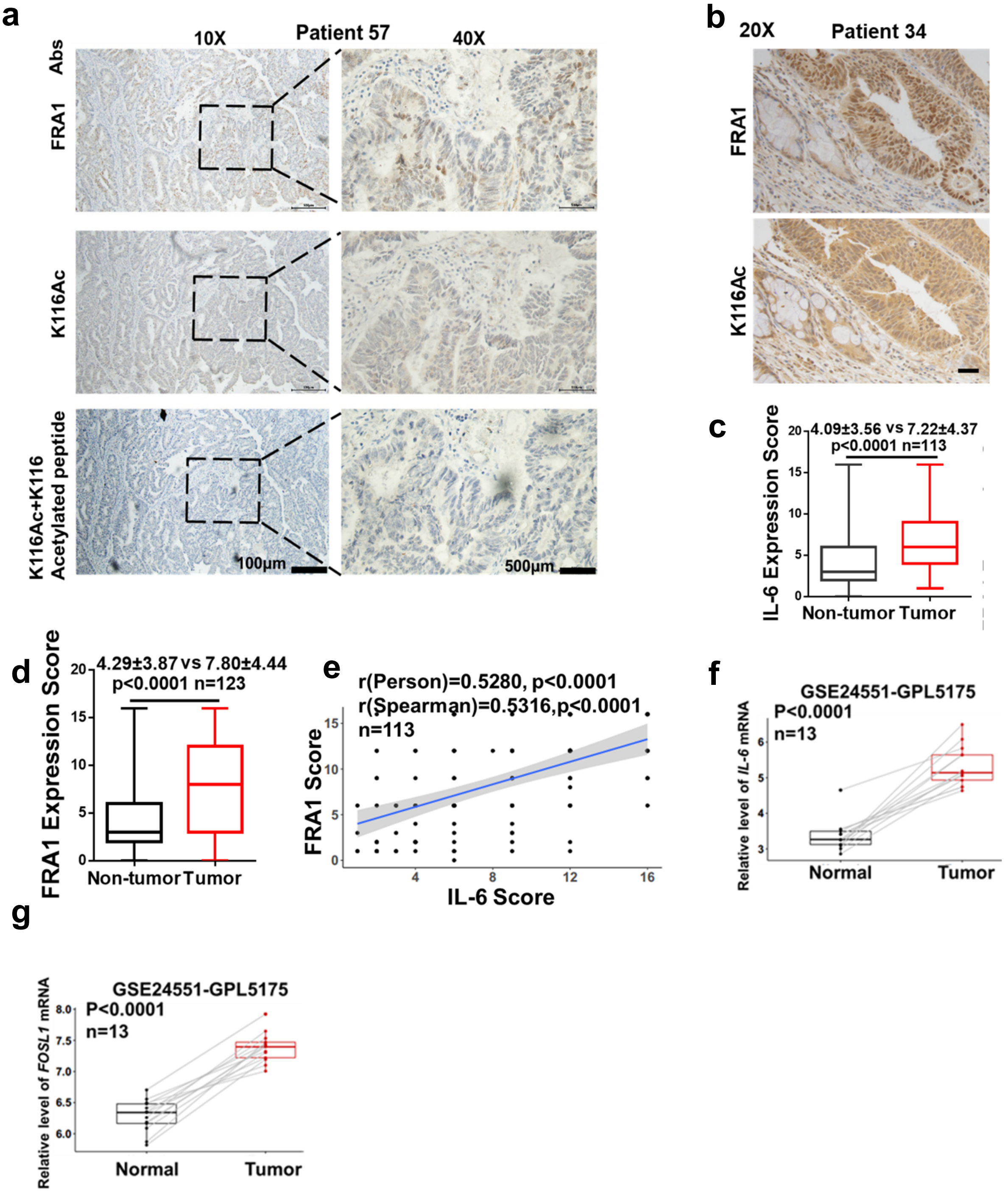

Figure S8. Combined treatment of 5-FU with the HDAC6 inhibitor Tubastatin A synergistically inhibits CRC stem-like properties and malignant growth

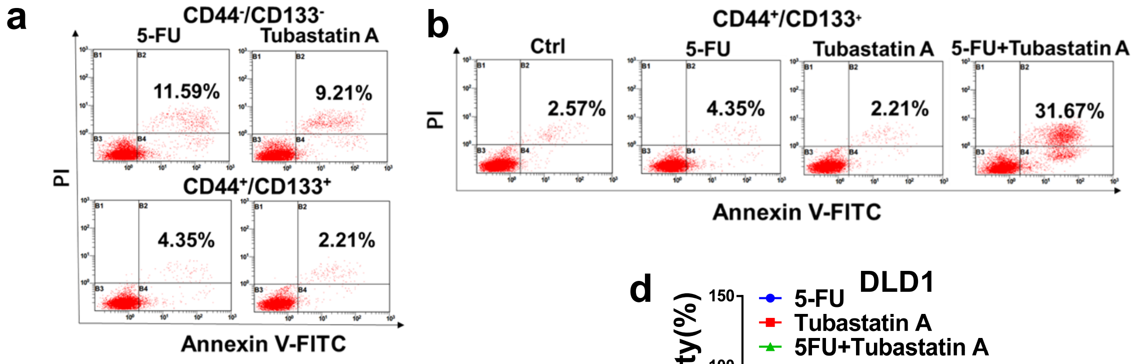

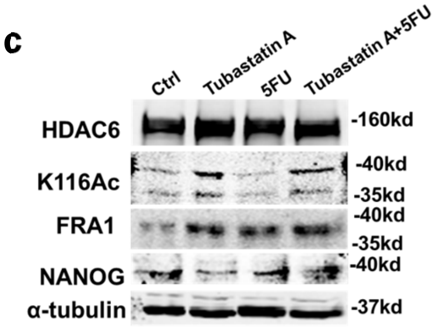

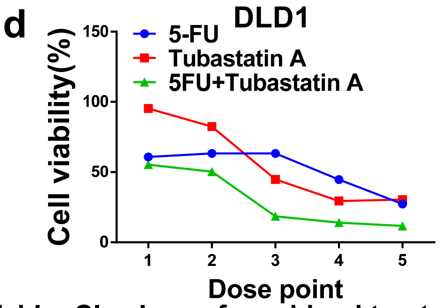

Table: CI values of combined treatment of 5-FU with Tubastatin A in DLD1 cell line

5-FU (uM)	Tuba (uM)	Fa	CI	Type of interaction
5	15	0.4468	0.532	Synergistic
10	30	0.4972	0.799	Synergistic
20	60	0.8139	0.304	Synergistic
40	120	0.8593	0.455	Synergistic
80	240	0.8827	0.767	Synergistic

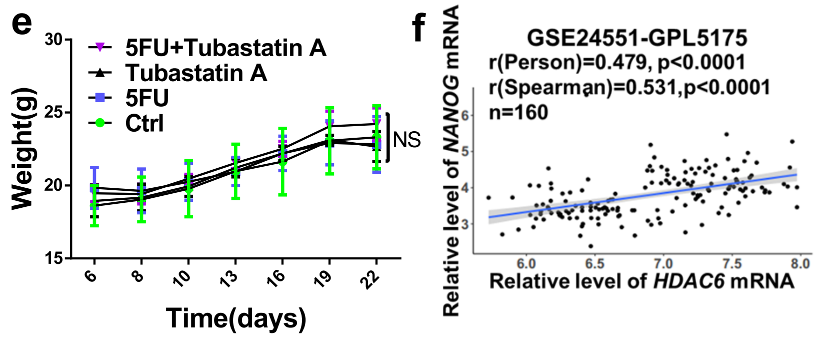

Supplement: Supplementary file 3 — supplementary figures [file 41388_2019_763_MOESM3_ESM.pdf]
